# Renal-Cardiac Crosstalk in the Pathogenesis and Progression of Heart Failure

**DOI:** 10.1161/CIRCRESAHA.124.325488

**Published:** 2025-05-23

**Authors:** Heidi Noels, Emiel P.C. van der Vorst, Sébastien Rubin, Amber Emmett, Nikolaus Marx, Maciej Tomaszewski, Joachim Jankowski

**Affiliations:** Institute for Molecular Cardiovascular Research (H.N., E.P.C.v.d.V., J.J.), Uniklinik RWTH Aachen, RWTH Aachen University, Germany.; Aachen-Maastricht Institute for Cardiorenal Disease (H.N., E.P.C.v.d.V., J.J.), Uniklinik RWTH Aachen, RWTH Aachen University, Germany.; Biochemistry Department (H.N.), Cardiovascular Research Institute Maastricht, Maastricht University, the Netherlands.; Pathology Department (J.J.), Cardiovascular Research Institute Maastricht, Maastricht University, the Netherlands.; Interdisciplinary Center for Clinical Research (IZKF) (E.P.C.v.d.V.), RWTH Aachen University, Germany.; Department of Internal Medicine I-Cardiology, Angiology and Internal Intensive Care Medicine (N.M.), RWTH Aachen University, Germany.; Institute for Cardiovascular Prevention, Ludwig-Maximilians-University Munich, Germany (E.P.C.v.d.V.).; L’Institut national de la santé et de la recherche médicale (INSERM), BMC, U1034, University of Bordeaux, Pessac, France (S.R.).; Renal Unit, University Hospital of Bordeaux, France (S.R.).; Faculty of Medicine, Biology and Health, Division of Cardiovascular Sciences, The University of Manchester, United Kingdom (A.E., M.T.).; British Heart Foundation Manchester Centre of Research Excellence, United Kingdom (M.T.).; Manchester Academic Health Science Centre, Manchester University National Health Service (NHS) Foundation Trust, United Kingdom (M.T.).; Signature Research Programme in Health Services and Systems Research, Duke-National University of Singapore (M.T.).

**Keywords:** cardiovascular diseases, heart failure, hypertension, inflammation, metabolism

## Abstract

Chronic kidney disease (CKD) represents a global health issue with a high socioeconomic impact. Beyond a progressive decline of kidney function, patients with CKD are at increased risk of cardiovascular diseases, including heart failure (HF) and sudden cardiac death. HF in CKD can manifest both as HF with reduced ejection fraction and HF with preserved ejection fraction, with the latter further increasing in relative importance in the more advanced stages of CKD. Typical cardiac remodeling characteristics in uremic cardiomyopathy include left ventricular hypertrophy, myocardial fibrosis, cardiac electrical dysregulation, capillary rarefaction, and microvascular dysfunction, which are triggered by increased cardiac preload, cardiac afterload, and preload and afterload-independent factors. The pathophysiological mechanisms underlying cardiac remodeling in CKD are multifactorial and include neurohormonal activation (with increased activation of the renin-angiotensin-aldosterone system, the sympathetic nervous system, and mineralocorticoid receptor signaling), cardiac steroid activation, mitochondrial dysfunction, inflammation, innate immune activation, and oxidative stress. Furthermore, disturbances in cardiac metabolism and calcium homeostasis, macrovascular and microvascular dysfunction, increased cellular profibrotic responses, the accumulation of uremic retention solutes, and mineral and bone disorders also contribute to cardiovascular disease and HF in CKD. Here, we review the current knowledge of HF in CKD, including the clinical characteristics and pathophysiological mechanisms revealed in animal studies. We also elaborate on the detrimental impact of comorbidities of CKD on HF using hypertension as an example and discuss the clinical characteristics of hypertensive heart disease and the genetic predisposition. Overall, this review aims to increase the understanding of HF in CKD to support future research and clinical translational approaches for improved diagnosis and therapy of this vulnerable patient population.

Chronic kidney disease (CKD) has become a significant global health issue, affecting millions of individuals worldwide. The prevalence of CKD is still rising, driven by an aging population and an increasing incidence of diabetes, hypertension, and other risk factors such as obesity and cardiovascular diseases (CVDs).^1^ Global health surveys and studies by the World Health Organization show that CKD affects ≈10% to 15% of adults. The GBD study (Global Burden of Disease) reports that over 850 million people are currently living with some form of CKD, making it more prevalent than cancer and CVD combined.^2^

The prevalence of CKD varies across different regions, reflecting variations in access to health care, genetics, and risk factors such as hypertension and diabetes. For instance, high-income countries, such as the United States, report a CKD prevalence of around 14% to 16%.^3^ East Asia and South Asia have similarly high rates, partly driven by the rising incidence of diabetes in these regions.^4^ In contrast, sub-Saharan Africa and Latin America report a lower prevalence but higher rates of progression to the end stage of kidney disease, primarily due to limited access to health care and treatment.^5^

The Kidney Disease: Improving Global Outcomes guidelines^6^ classify CKD into 5 stages by evaluating kidney function by the glomerular filtration rate (GFR) and detecting kidney damage by albuminuria: CKD can progress from a stage with normal kidney filtration function but the presence of kidney damage (CDK stage 1) to a stage with mildly, moderately, or severely reduced kidney function (CKD stages 1–3). In stage 5 (GFR <15 mL/min) or end-stage renal disease (ESRD), patients need dialysis (CKD5D) or a kidney transplant for survival (Figure 1).

**Figure 1.**
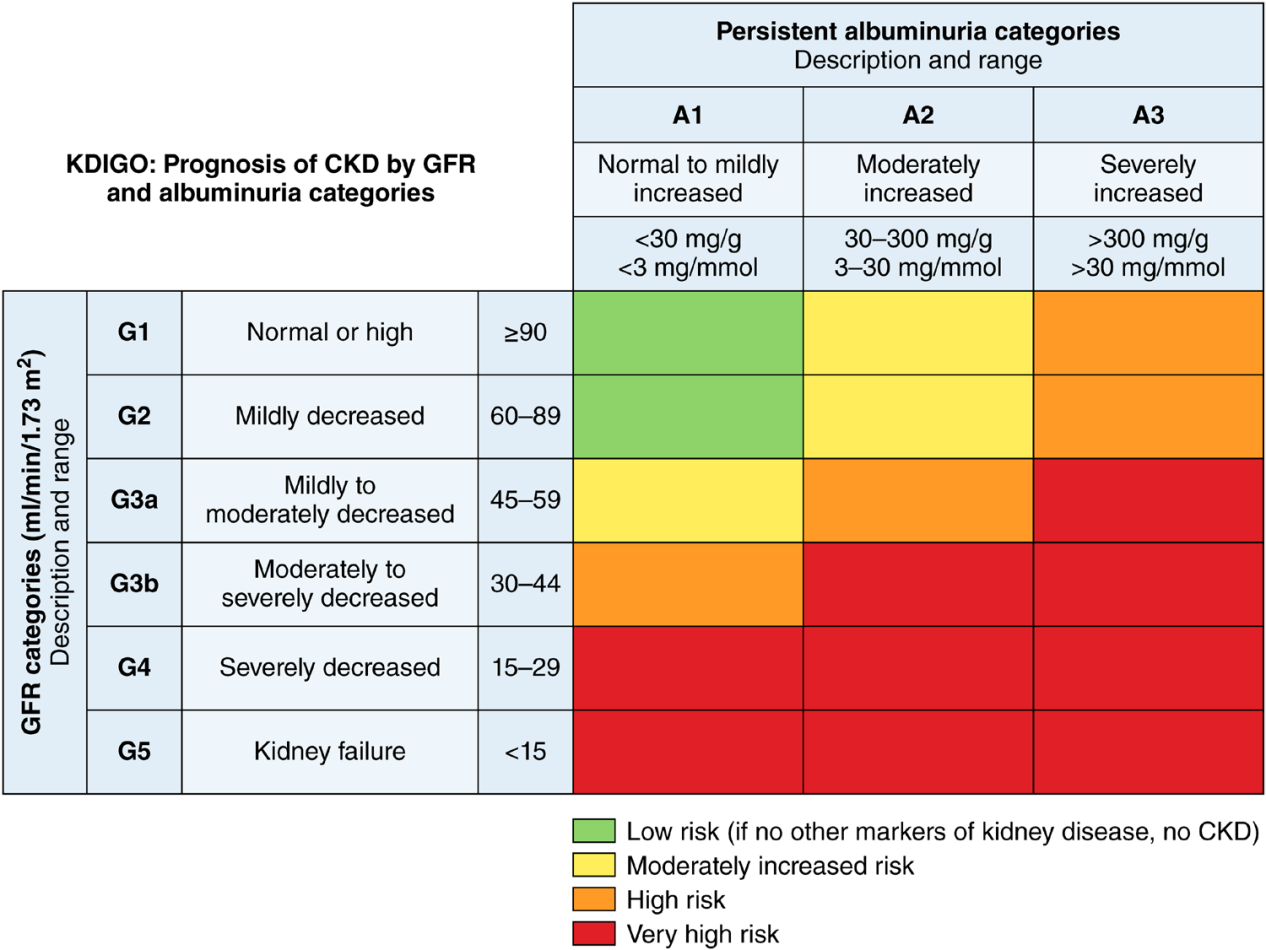
**KDIGO (Kidney Disease Improving Global Outcomes) classification of chronic kidney disease (CKD) from the 2024 KDIGO guidelines.** The table visualizes CKD classification based on glomerular filtration rate (GFR) category (G1–G5) and albuminuria category (A1–A3). Illustration credit: Sceyence Studios. Adapted from the work in reference ^6^ with permission from Elsevier.

Because albuminuria and GFR are key in classifying CKD, the Kidney Disease: Improving Global Outcomes guidelines also assess its severity using the albumin-to-creatinine ratio (ACR).^6^ The combination of GFR and albuminuria based on the Kidney Disease: Improving Global Outcomes guidelines is used to determine the severity of CKD and guide clinical decision-making (Figure 1). Patients with lower GFR and higher albuminuria levels face a greater risk of progressing to the end stage of kidney disease but also show increased cardiovascular complications and increased mortality.

Of note, both reduced GFR and increased albuminuria increase the risk of all-cause and cardiovascular death independent of traditional risk factors^7–9^ (Figure 2). While CVD is responsible for mortality in 21.7% to 26.0% of the general population, this is increased to 33.3% to 37.1% in patients with CKD stage 3 and to 39.9% in patients with CKD stage 4.^10^ Patients on hemodialysis revealed CVD mortality rates that were 10× to 20× higher compared with the general population or even up to ≈400 times higher for patients aged 25 to 34 years.^11^ Overall, CVD presents in 63.4%, 66.6%, and 75.3% of patients with CKD whose kidney dysfunction is, respectively, mild (CKD stages 1 and 2), moderate (CKD stage 3), and severe or even complete (CKD stages 4 and 5D) compared with 37.5% in individuals with healthy kidney function^12^ (Figure 3A).

**Figure 2.**
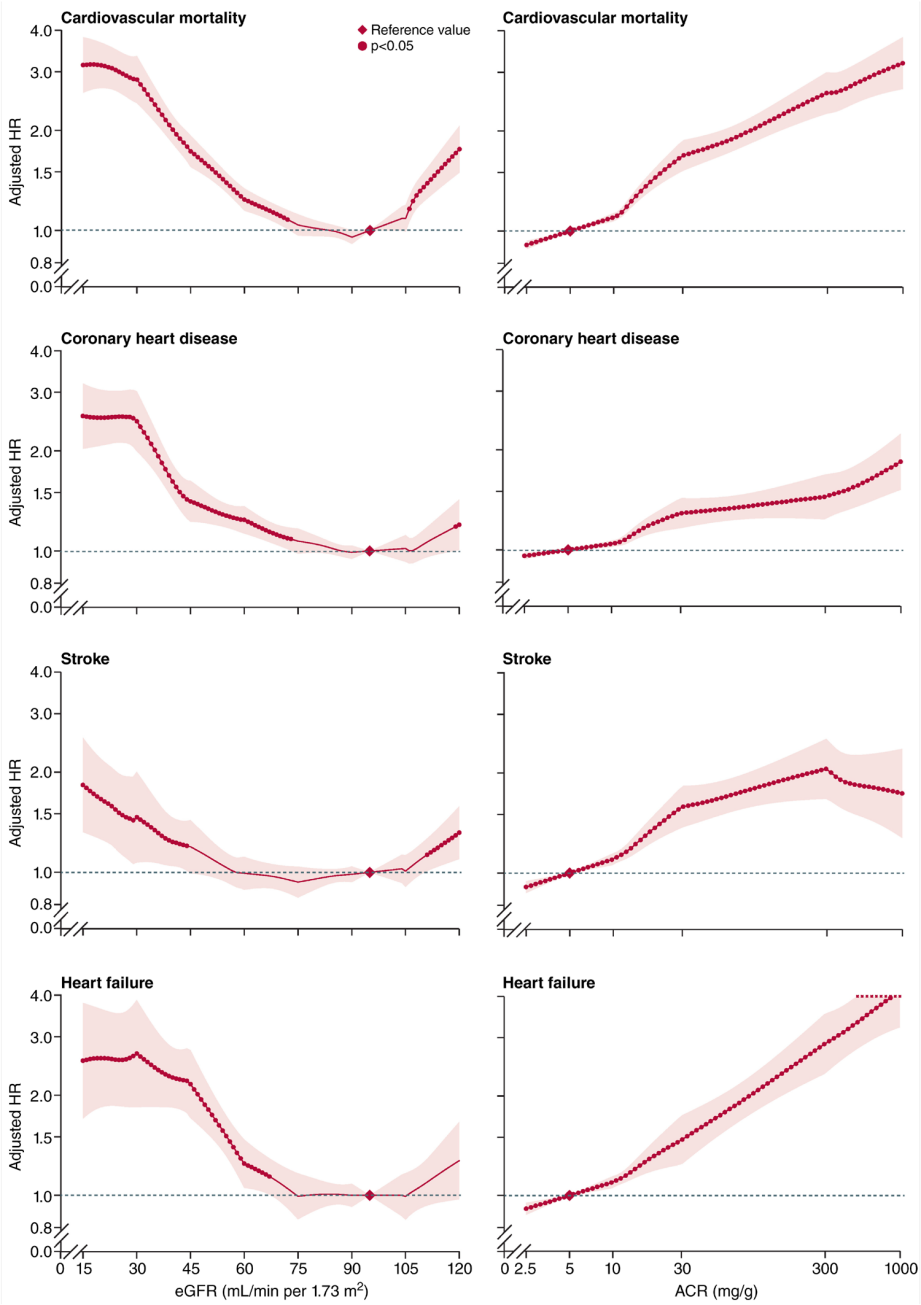
**Increased cardiovascular risk in patients with chronic kidney disease (CKD).** Adjusted hazard ratios (HRs) and 95% CIs (shaded areas or whisker plots) of cardiovascular mortality (top row), coronary heart disease (second row), stroke (third row), and heart failure (bottom row) in function of estimated glomerular filtration rate (eGFR; **left**) and albumin-to-creatinine ratio (ACR; **right**), combining the combined general population and high-risk cohorts within the database of the CKD Prognosis Consortium. The analysis integrated data from 24 cohorts with data on fatal and nonfatal cardiovascular outcomes and a median follow-up time >4 years. Reference (diamond): eGFR of 95 mL/min per 1.73 m^2^ and ACR of 5 mg/g. Dots represent statistical significance (*P*<0.05). *Adjustments were for age, sex, race/ethnicity, smoking, systolic blood pressure, antihypertensive drugs, diabetes, total and high-density lipoprotein cholesterol concentrations, and albuminuria (ACR or dipstick) or eGFR, as appropriate. For analyses of eGFR: 629 776 participants for cardiovascular mortality, 144 874 for coronary heart disease, 137 658 for stroke, and 105 127 for heart failure. For analyses of ACR: 120 148 participants for cardiovascular mortality, 91 185 for coronary heart disease, 82 646 for stroke, and 55 855 for heart failure. Illustration credit: Sceyence Studios. Adapted from Matsushita et al^9^ with permission from Elsevier.

**Figure 3.**
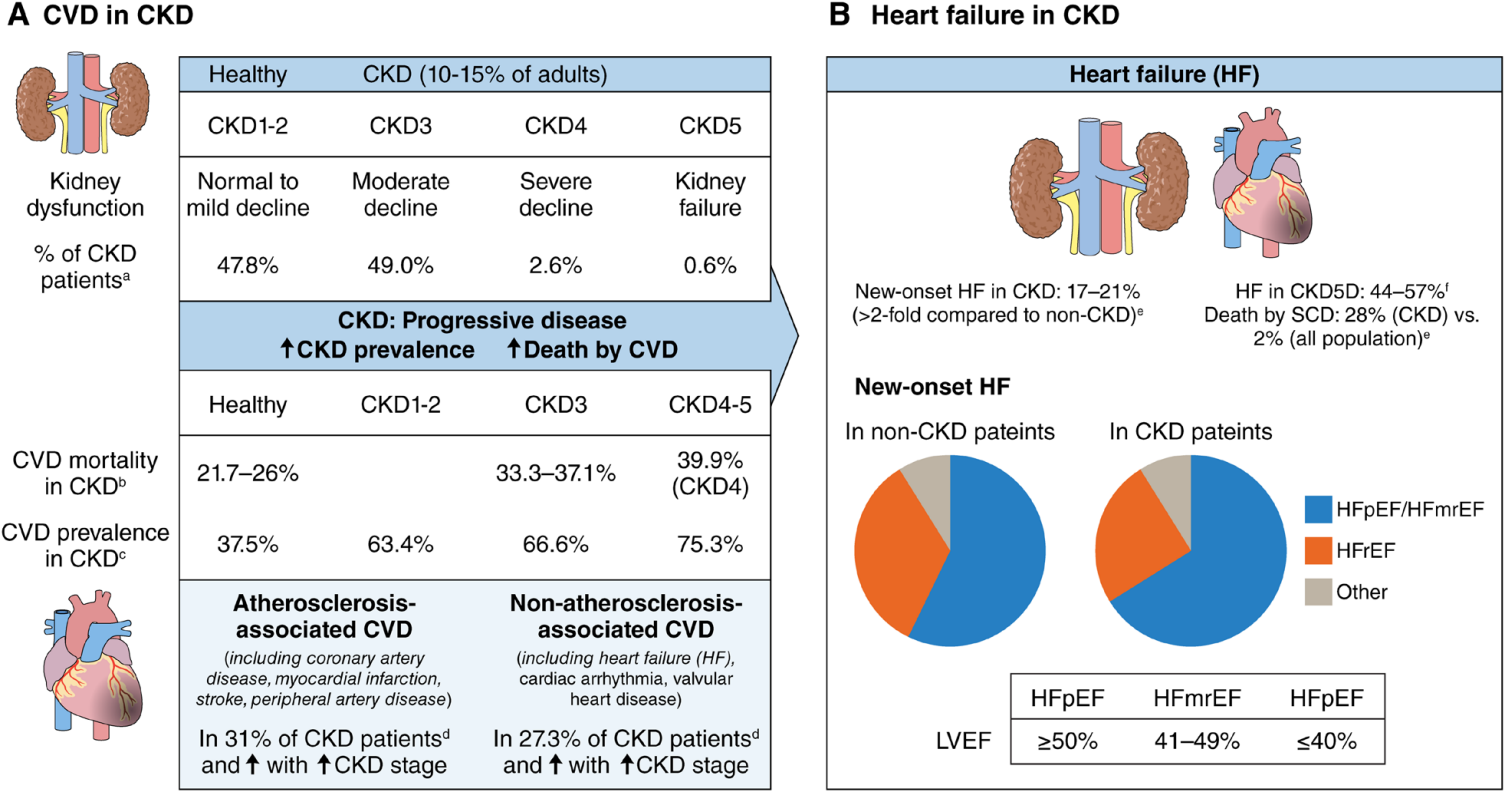
**Increased risk of cardiovascular disease (CVD) and heart failure (HF) in chronic kidney disease (CKD). A**, CKD is a progressive disease and is classified in stages CKD1-5 based on glomerular filtration rate. Patients with CKD show an increased prevalence of CVD and an increased risk of CVD-associated mortality. Increased CVD risk in CKD includes both atherosclerosis-associated CVD and nonatherosclerosis-associated CVD, such as HF. **B**, HF and sudden cardiac death are increased in patients with (advanced) CKD, with a contribution of both HF with reduced ejection fraction (HFrEF) and heart failure with preserved ejection fraction (HFpEF). **A** and **B**, Data based on a,^13^ b,^10^ c,^12^ d,^14^ e,^15,16^ f,^17,18^ and g.^19^ Illustration credit: Sceyence Studios. HFmrEF indicates HF with mildly reduced ejection fraction; and LVEF, left ventricular ejection fraction.

Both atherosclerosis-associated CVD (atheromatous CVD, including coronary artery disease, myocardial infarction (MI), stroke, and peripheral artery disease) and nonatherosclerosis-associated CVD (nonatheromatous CVD, including heart failure [HF], cardiac arrhythmia, and valvular heart disease) are highly prevalent in patients with CKD with 31.0% and 27.3%, respectively,^14^ and increase in prevalence with CKD progression^9,14^ (Figure 2). Furthermore, above 65 years of age, patients with advanced CKD have an increased risk of being diagnosed with >1 type of CVD.^14^ Concerning mortality, death by MI and stroke remains high in patients with CKD. Still, compared with the general population, there is primarily an increase in mortality due to sudden cardiac death (28% versus 2%) and HF (9% versus 7%).^19^ According to left ventricular (LV) ejection fraction, HF is classified as HF with reduced ejection fraction (HFrEF; LV ejection fraction ≤40%), HF with mildly reduced ejection fraction (LV ejection fraction: 41%–49%), and HF with preserved ejection fraction (HFpEF; LV ejection fraction ≥50%).^20^ Sudden cardiac death underlies a large proportion of deaths in HF, representing 30% to 50% of mortality in patients with HFrEF^21^ and 40% of cardiovascular deaths (25% of all deaths) in HFpEF.^22^

Here, we focus on increased HF risk in CKD, the clinical characteristics, underlying pathophysiological mechanisms, and molecular mediators as revealed in animal studies. This also includes a discussion of uremic retention solutes that gradually accumulate in patients with CKD due to declining kidney function, in part combined with increased production. These uremic retention solutes comprise a range of molecules that, at elevated concentrations as found in patients with CKD, can induce pathophysiological effects on the body and are, hence, referred to as uremic toxins. As one class, protein-bound uremic toxins comprise hydrophobic molecules that bind proteins in the blood and form higher molecular weight complexes that can hardly be removed during dialysis, resulting in high concentrations in patients in the end stage of CKD despite regular dialysis. Beyond providing clinical and mechanistic insights into HF in CKD, the impact of hypertension as the most common comorbidity of both CKD and HF is discussed from a clinical point of view.

## Increased Risk of HF in Patients With CKD

Around half of patients with HF also suffer from CKD.^23^ Conversely, patients with CKD present more often with HF compared with those without CKD and display new-onset HF in the range of 17% to 21%,^15,16^ with the age-adjusted incidence rate of new-onset HF being more than doubled in patients with CKD3 or higher compared with patients without CKD.^15^ Overall, ≈44% to 57% of patients with CKD on hemodialysis have HF.^17,18^ Beyond copresentation, CKD and HF mutually influence disease outcomes. Mortality risk was more than doubled in patients with HF with CKD compared with those without CKD, and severe kidney impairment was a predictor of mortality in HF.^23^ Conversely, HF also increases the risk of CKD development and progression.^24^

Whereas, overall, patients with HFrEF have a higher prevalence of ischemic heart disease (≈two-thirds of cases), patients with HFpEF are mostly older and show a lower prevalence of cardiac ischemia (≈25%) but a higher coexistence of hypertension, atrial fibrillation, CKD, obesity, and type 2 diabetes.^16,25^ Patients hospitalized with new-onset HF present more frequently with preserved or mildly reduced ejection fraction (HFpEF/HF with mildly reduced ejection fraction) than reduced ejection fraction (HFrEF), which was a bit more pronounced in patients with CKD (66% [58%] versus 25% [35%]) compared with those without CKD (57% [54%] versus 34% [38%]; values presented as crude or adjusted percentages, respectively, after adjustment for age, sex, race, obesity, diabetes, hypertension, atrial fibrillation, and MI)^26^ (Figure 3B). HFpEF was found to be even more prevalent in hospitalized patients with new-onset HF with severe CKD compared with those with mild CKD (CKD5D: 70% versus CKD1-2: 54%).^26^ Similar findings of increased presentation of HFpEF over HFrEF in CKD were reported when analyzing incident HF in 3557 patients with mild to severe CKD from the CRIC (Chronic Renal Insufficiency) cohort,^27^ as well as upon analysis of a cohort of 214 patients with CKD on chronic hemodialysis from 5 hemodialysis units presenting with HF.^18^ In the latter study, most hemodialysis patients with HF presented with HFpEF (61% versus HFmEF: 12%; HFrEF: 9%; and other HF: 18%).^18^ The association of incident HF with mortality rates was higher in patients with CKD with HFrEF compared with HFpEF (HFrEF: hazard ratio, 2.73 [95% CI, 2.24–3.33] versus HFpEF: hazard ratio, 1.99 [95% CI, 1.65–2.40]; *P*=0.0002) though without significant differences in HF association with progression to CKD5D.^27^

**Figure 4.**
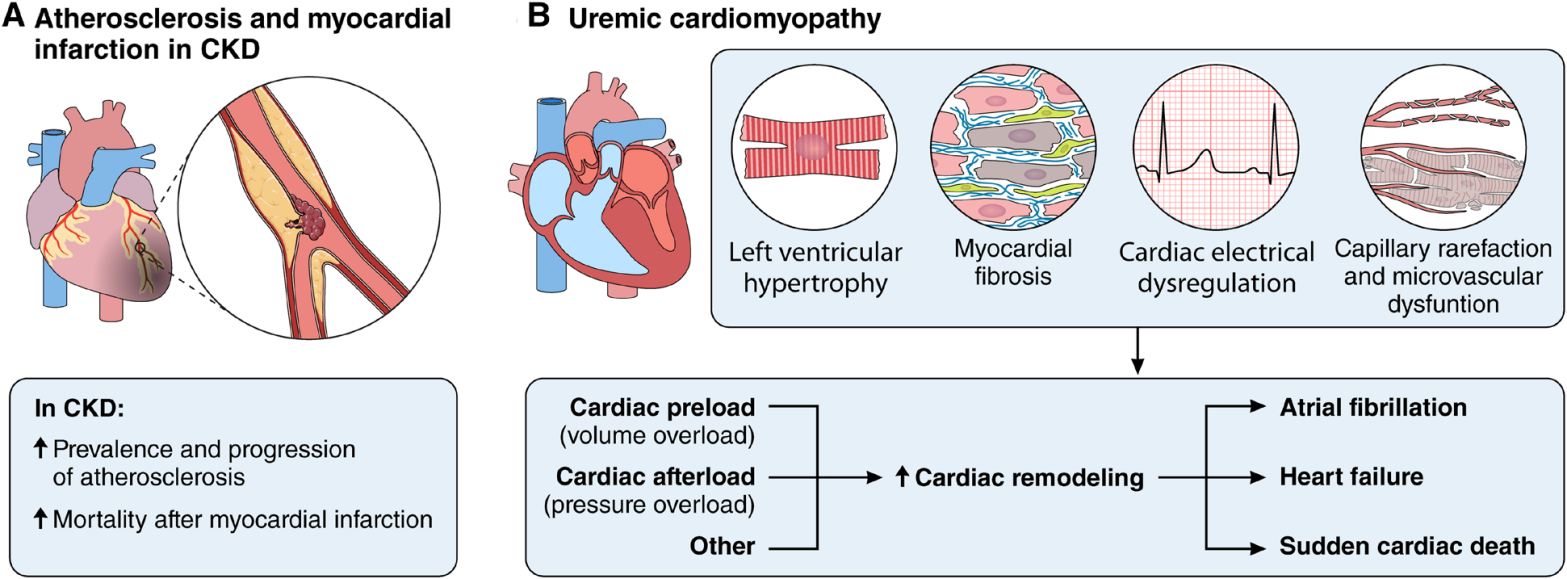
**Increased risk of atherosclerosis-associated and nonatherosclerosis-associated cardiovascular disease (CVD) in chronic kidney disease (CKD). A**, Patients with CKD display an increased prevalence and progression of atherosclerosis and increased mortality after myocardial infarction. **B**, Patients with CKD display features of cardiac remodeling, referred to as uremic cardiomyopathy: characteristics, causal factors, and consequences. For more information, see text. Illustration credit: Sceyence Studios.

Atherosclerosis is the leading underlying cause of ischemic heart disease, MI, and associated HF, and both the prevalence and the progression of atherosclerosis are increased in CKD.^28^ Furthermore, after MI, patients with CKD show a higher mortality rate (23%) compared with patients without CKD (12.6%),^29^ which worsens with a more advanced CKD stage^30^ (Figure 4A).

Patients with CKD also present with nonischemic cardiac remodeling, referred to as uremic cardiomyopathy^31^ (Figure 4B). Typical characteristics include LV hypertrophy, myocardial fibrosis, and capillary rarefaction, contributing to increased HF risk.^31^ Also, electric alterations occur, including atrial fibrillation,^31–33^ which is interlinked in a vicious cycle with HF.^34^ An increase in cardiac preload (volume overload) and afterload (pressure overload) in patients with CKD contributes to cardiac remodeling in CKD. Triggers of increased afterload include arterial stiffness, microvascular dysfunction, aortic stenosis, and systemic hypertension, all of which are increased in patients with CKD.^35,36^ Furthermore, additional factors independent of increased cardiac preload and afterload contribute to uremic cardiomyopathy and associated HF, as revealed in animal studies and discussed in more detail in the following.

## Cardiovascular Remodeling and Systemic Alterations Underlying HF in CKD: Clinical Observations

Uremic cardiomyopathy and associated HF are characterized by several local and systemic alterations, such as LV hypertrophy, cardiac fibrosis, cardiac electrical disturbance, vascular dysfunction, low-grade chronic inflammation, innate immune activation, and enhanced neurohormonal signaling, which will be discussed in detail here.

### LV Hypertrophy and Cardiac Fibrosis

Around 30% to 40% of patients with kidney disease also have LV hypertrophy, while the prevalence of LV hypertrophy rises tremendously from 70% to 90% upon kidney failure.^37^ Therefore, LV hypertrophy is the most prominent cardiovascular risk marker in patients with CKD.^38–40^ LV hypertrophy is characterized as a thickening of the LV wall, which coincides with an increase in LV mass.^41^ This hypertrophy develops as an adaptive mechanism to regulate vascular wall stress due to pressure or volume overload.^19,42^ Thereby, there are 2 major forms of LV hypertrophy: concentric hypertrophy, which results from pressure overload, resulting in the parallel arrangement of contractile protein units,^43^ and eccentric hypertrophy, which results from volume overload, resulting in the lengthening of contractile units.^43,44^ Although LV hypertrophy initially functions adaptively, over time, it becomes a pathological maladaptation enhancing cardiomyocyte death, further progressing cardiomyopathy.^43,45,46^ Persistent cardiac hypertrophy can eventually lead to HF and sudden death.^47,48^

Besides the macroscopic LV hypertrophy, several microscopic features are also observed in patients with CKD, particularly myocardial fibrosis. Several clinical and autopsy studies indeed revealed that increased cardiac fibrosis is present in patients with CKD compared with those without CKD (reviewed in the study by Junho et al^31^). Furthermore, several factors that may be responsible for the interaction between CKD and myocardial fibrosis have already been identified although causality still needs to be proven.^49^

### Cardiac Electrical Disturbance

The risk of atrial and ventricular arrhythmias is inversely correlated with kidney function. In an analysis of the ARIC (Atherosclerosis Risk in Community Study), it could, for example, be shown that >90% of patients with GFR < 60 mL/min per m^2^ have ectopic beats, while 30.2% have ventricular tachycardia and 7.4% have atrial fibrillation.^50^ Strikingly, around one-third of mortality in dialysis patients is caused by atrial arrhythmias.^51,52^

Various factors can cause atrial arrhythmias in patients with CKD. For example, inflammation-causing fibrosis and hypertrophy activate cardiac fibroblasts, nonexcitable cells that can conduct electrical currents.^53^ This causes the depolarization of myocytes, resulting in arrhythmias.^54^ In addition, activation of the renin-angiotensin-aldosterone system (RAAS) upon kidney damage results in the retention of large amounts of sodium and water and, thus, volume overload.^55^ As described above, this causes structural changes, that is, fibrosis and hypertrophy, resulting in arrhythmias. The accumulation of uremic toxins can also cause structural changes that lead to a disruption of electrical conductivity.^56^

Electrolyte disbalances can also play a crucial role in the development of arrhythmias. For example, hyperkalemia is often observed in patients with severe CKD on dialysis due to imbalances in its intake and removal. This hyperkalemia subsequently triggers hyperpolarization of myocytes and, thereby, causes disturbances in the cardiac conductivity.^57^

### Vascular Dysfunction

Besides the above-described local alterations, CKD and HF are characterized by several systemic changes. For example, patients with CKD display increased macrovascular and microvascular dysfunction, as we recently discussed in detail elsewhere.^35^ On the level of the macrovasculature, endothelial dysfunction, impaired vasorelaxation, and enhanced vascular stiffness contribute to increased cardiovascular risk in patients with and without CKD.^35^ In patients with HF, endothelial dysfunction in the peripheral conduit arteries (measured based on flow-mediated dilation) increased the risk of future cardiac events and mortality.^58–60^ Furthermore, a dysfunction of the microvasculature, which includes the arterioles, capillaries, and venules enabling tissue perfusion, also increases the risk of cardiovascular events in the general population and in patients with high cardiovascular risk.^35^ Microvascular dysfunction encompasses a functional deterioration reflected by reduced vasodilatory responses. It also includes structural maladaptive alterations, such as microvascular rarefaction, a luminar narrowing triggered by vascular remodeling or perivascular fibrosis, and microembolization.^61–64^ In HFrEF, peripheral microvascular dysfunction was associated with increased HF-related events.^65^ Capillary rarefaction and microvascular dysfunction have also been observed in the hearts of patients with HFpEF.^66,67^ However, actual causal involvement in HF currently remains debated.^68,69^

### Low-Grade Chronic Inflammation and Innate Immune Activation

Another systemic alteration upon CKD is the development of chronic inflammation,^70,71^ characterized by increases in hs-CRP (high-sensitivity C-reactive protein), increased white blood cell count, and increases in cytokines such as IL-1β, IL-6, and TNFα (tumor necrosis factor alpha).^72–74^ This has been confirmed in a population-based cohort study of 5000 people with a 15-year follow-up.^74^ In the CRIC study focusing on patients with CKD, these inflammatory markers correlated with faster progression to kidney failure, development of atherosclerosis, and death.^72,73^ This is in line with results from a meta-analysis evaluating 54 cohort studies, encompassing 160 309 people, demonstrating that hs-CRP was a strong predictor for coronary heart disease and death.^75^ Furthermore, in patients with CKD stages 2 to 5, it could be shown that high serum IL-6 levels were associated with a history of CVD and even predicted the incidence rate of future cardiovascular events (hazard ratio, 1.66).^76^

Such inflammatory reactions are caused by cellular signaling processes, starting with activating surface receptors such as TLRs (toll-like receptors). Receptor activation subsequently triggers the activation of key regulatory proteins such as NF-κB (nuclear factor κB) and the NLRP3 (nucleotide-binding domain family pyrin domain containing 3) inflammasome.

Several clinical trials have also already been performed to validate the role of inflammation and provide potential therapeutic opportunities. A recent meta-analysis that included major clinical trials such as LoDoCo and COLCOT (Colchicine Cardiovascular Outcomes) demonstrated the efficacy of colchicine, an anti-inflammatory molecule that inhibits NLRP3 activation, in reducing cardiovascular events. Specifically, in patients with coronary disease, colchicine reduced the relative risk of MI by 22% and stroke by 46%.^77^ However, as a note of caution, there was a nonsignificant trend toward higher noncardiovascular mortality. In addition, although colchicine seems promising to reduce inflammation and CVD risk, most trials to date excluded patients with CKD because colchicine accumulates in these patients.^78^ Other trials have focussed on the NLRP3-dependent cytokines, such as the CANTOS (Canakinumab Anti‐Inflammatory Thrombosis Outcomes) trial, which tested a monoclonal antibody, canakinumab, against IL-1β in patients with CVD.^79^ Thereby, it could be shown that canakinumab also reduced hs-CRP and IL-6, resulting in a reduced risk for cardiovascular death, among others. Interestingly, this cardioprotective effect was also observed in a subgroup of patients with CKD stage 3, while no nephroprotective effect was observed.^79^ These observations also initiated studies directly targeting IL-6, for example, using a monoclonal antibody called ziltivekimab. The RESCUE (Randomized Evaluation of Patients with Stable Angina Comparing Utilization of Noninvasive Examinations) trial^80^ demonstrated sharp reductions in hs-CRP (77%–92%, dose-dependent) in patients with moderate-to-severe CKD, reflecting a much more substantial reduction than CANTOS (26%–41%). Currently, a large trial, ZEUS (Zlitivekimab Cardiovascular Outcomes Study), is ongoing to evaluate the effects of ziltivekimab in people with CVD, CKD, and inflammation,^81^ which will provide valuable outcomes for the effectiveness of IL-6 targeting in CVD and, particularly, patients with CKD.

### Enhanced Neurohormonal Signaling

Patients with advanced stages of CKD have a permanent status of fluid overload, resulting in hypertension in 60% to 90% of the patients depending on the CKD stage.^82,83^ This is caused by the continuous activation of the RAAS, the sympathetic nervous system (SNS), and mineralocorticoid receptor (MR) signaling.

The RAAS plays a key role in the pathogenesis of CKD, where, particularly, Ang (angiotensin) II acts as a central mediator.^84,85^ Therefore, inhibition of the RAAS by either ACE (angiotensin-converting enzyme) inhibitors or angiotensin receptor blockers (ARBs) has been demonstrated to be an effective treatment approach. For example, a meta-analysis of 12 763 patients with HFrEF demonstrated that ACE inhibitors significantly reduce total mortality, as well as lower rates of readmission for HF and lower incidence of MI.^86^ In addition, several clinical trials showed that ARB therapy also reduced mortality and hospitalization in patients with HF.^87,88^ However, the effects are mostly smaller than the ones observed for ACE inhibitors although ARBs are generally better tolerated. The benefits of RAAS inhibitors on HFpEF are less clear to date. Although some studies showed a nonsignificant trend toward a benefit with ACE inhibitors or ARBs,^89,90^ other studies did not show any effects on mortality or hospitalization,^91^ suggesting that RAAS inhibition’s effect on HFpEF is rather minimal.

Besides the RAAS, the SNS also plays a fundamental role in blood pressure control. CKD results in an overactivation of the β-adrenergic receptors by catecholamine neurotransmitters, which contributes to cardiovascular complications in patients with CKD.^92^ Therefore, β-blockers have been evaluated, which block this β-adrenergic receptor signaling. It could be shown in patients with HF that β-blocker treatment results in significant reductions in ventricular ectopic beats, ventricular arrhythmias, and sudden cardiac death.^93,94^ More recently, renal denervation, in which efferent and afferent nerves connecting the kidney with the brain are modulated, also showed beneficial effects. For example, renal denervation resulted in a striking lowering of blood pressure in resistant hypertensive patients, coinciding with a sharp decrease in sympathetic nerve activity.^95^ To date, renal denervation has been proven safe in various studies and especially beneficial in hypertensive patients with CKD,^96–98^ as also nicely reviewed in the study by Kiuchi et al.^99^ While the effects of renal denervation and the resulting blood pressure lowering on CVD outcomes remain somewhat elusive, one study assessed the impact of renal denervation on cardiovascular outcomes and identified that renal denervation in patients with uncontrolled hypertension resulted in significant risk reductions in major cardiovascular events.^100^ However, larger clinical trials are required to confirm these beneficial effects on CVD outcomes. However, it has also been debated whether blood pressure can be used as a surrogate for outcome data.^101^

While MR signaling is essential as a regulator of electrolyte and fluid homeostasis, overactivation has detrimental effects on the kidney and the heart by contributing to inflammation and fibrosis.^102,103^ In line with this, MR antagonists exert anti-inflammatory and antifibrotic effects and are a promising therapeutic approach, as reviewed in detail in the study by Capelli et al.^104^ The RALES (Randomized Aldactone Evaluation Study) trial was the first large trial highlighting the impact of the MR antagonist spironolactone in patients with HFrEF.^105^ It could be shown that spironolactone treatment resulted in a large reduction in mortality rate in patients with HFrEF. A large meta-analysis of 15 clinical trials evaluating the effects of MR antagonists (spironolactone and eplerenone) demonstrated a good nephroprotective effect as they showed a marked reduction of proteinuria although hyperkalemia was a major side effect that still regularly occurred.^106^ A recent study pooled patient data from the RALES and EMPHASIS-HF (Eplerenone in Mild Patients Hospitalization and Survival Study in Heart Failure) trials, including 4355 patients with HF, to study the association between MR antagonist treatment and cardiovascular death or HF hospitalization.^107^ Overall, MR antagonist treatment reduced the risk of these primary outcomes. Interestingly, the risk reduction in primary outcome with MR antagonist therapy was similar in patients who experienced a deterioration of estimated GFR (eGFR) compared with people without eGFR reduction, demonstrating that a decline in eGFR should not automatically result in a discontinuation of treatment.^107^ Due to the risk of hyperkalemia, new nonsteroidal molecules have been developed to reduce this risk, for example, apararenone, esaxerenone, finerenone, and apararenone. While several clinical trials are ongoing, others have already proven that at least the same efficacy can be achieved with reduced risk, as reviewed in the study by Capelli et al.^104^

## Mechanisms Underlying Increased Cardiac Remodeling and HF in CKD: Insights From Animal Models

### Animal Models of Uremic Cardiomyopathy and HF in CKD

Animal models with CKD and signatures of cardiac fibrosis, LV hypertrophy, inflammation, cardiac electrical alterations, and cardiac systolic and diastolic dysfunction have contributed to a better understanding of the causal factors underlying cardiac remodeling and HF in CKD.^31,108^ These models induced CKD by subtotal nephrectomy, kidney ischemia/reperfusion injury, or adenine-, oxalate-, or cisplatin-induced kidney injury to study the impact of chronic kidney damage on cardiac remodeling. This was studied either in single-hit models or in multifactor hit models, which combined kidney injury with additional cardiovascular risk factors, such as hyperphosphatemia, hypertension, hyperlipidemia, and diabetes, to better mimic the common multimorbid character of patients with CKD.^31,108^

Overall, inducing uremic cardiomyopathy has proven challenging particularly in mice, with variable effects observed of CKD on cardiac remodeling and dysfunction. As we recently summarized in a systematic review with meta-analysis^108^ (and updated in the study by Junho et al^31^), several factors may influence these outcomes, including the mouse strain, the method of CKD induction, existing comorbidities such as hypertension or obesity, and the specific parameters used to assess cardiac remodeling and cardiac dysfunction. In short, a more consistent induction of uremic cardiomyopathy with signatures of cardiac hypertrophy, fibrosis, and dysfunction has been observed in 129/Sv mice compared with C57BL/6 mice.^31,108^ Compared with C57BL/6 mice, 129/Sv mice express 2 instead of 1 renin gene and more consistently develop increased blood pressure upon CKD induction. Overall, alterations in hypertension and RAAS signaling, as well as blood pressure–independent effects, triggered by genetic variances are thought to contribute to the mouse strain–dependent differences in the development of CKD-induced cardiomyopathy.^31,108^ Furthermore, rat models with CKD induction by subtotal nephrectomy or an adenine-rich diet have been shown to develop signatures of uremic cardiomyopathy, including cardiac hypertrophy, fibrosis, and cardiac dysfunction.^31^

Compared with single-hit models, the parallel induction of CKD with additional CKD comorbidities in multifactorial hit models increased the susceptibility to organ damage. It more consistently triggered the development of cardiac hypertrophy and fibrosis in CKD conditions.^31,108^

Hypertension is an important comorbidity of CKD and is both a consequence of and a contributor to chronic kidney damage. Hypertension also contributes to the development of CVD, including vascular dysfunction and remodeling, as well as cardiac remodeling with cardiac fibrosis, hypertrophy, and hypertensive heart disease. Animal models of persistent hypertension have provided insights into the pathophysiological mechanisms that link hypertension to kidney and cardiovascular damage, as discussed in detail elsewhere.^109^ In the context of CKD, animal models combining CKD with additional hypertension-inducing strategies more consistently developed clinical signatures of uremic cardiomyopathy compared with single-hit CKD models.^31,108^ In addition to hypertension, also hyperphosphatemia, hyperlipidemia, and diabetes have been combined with CKD to study the impact of the multimorbid condition, as observed in patients with CKD, on the heart.

**Figure 5.**
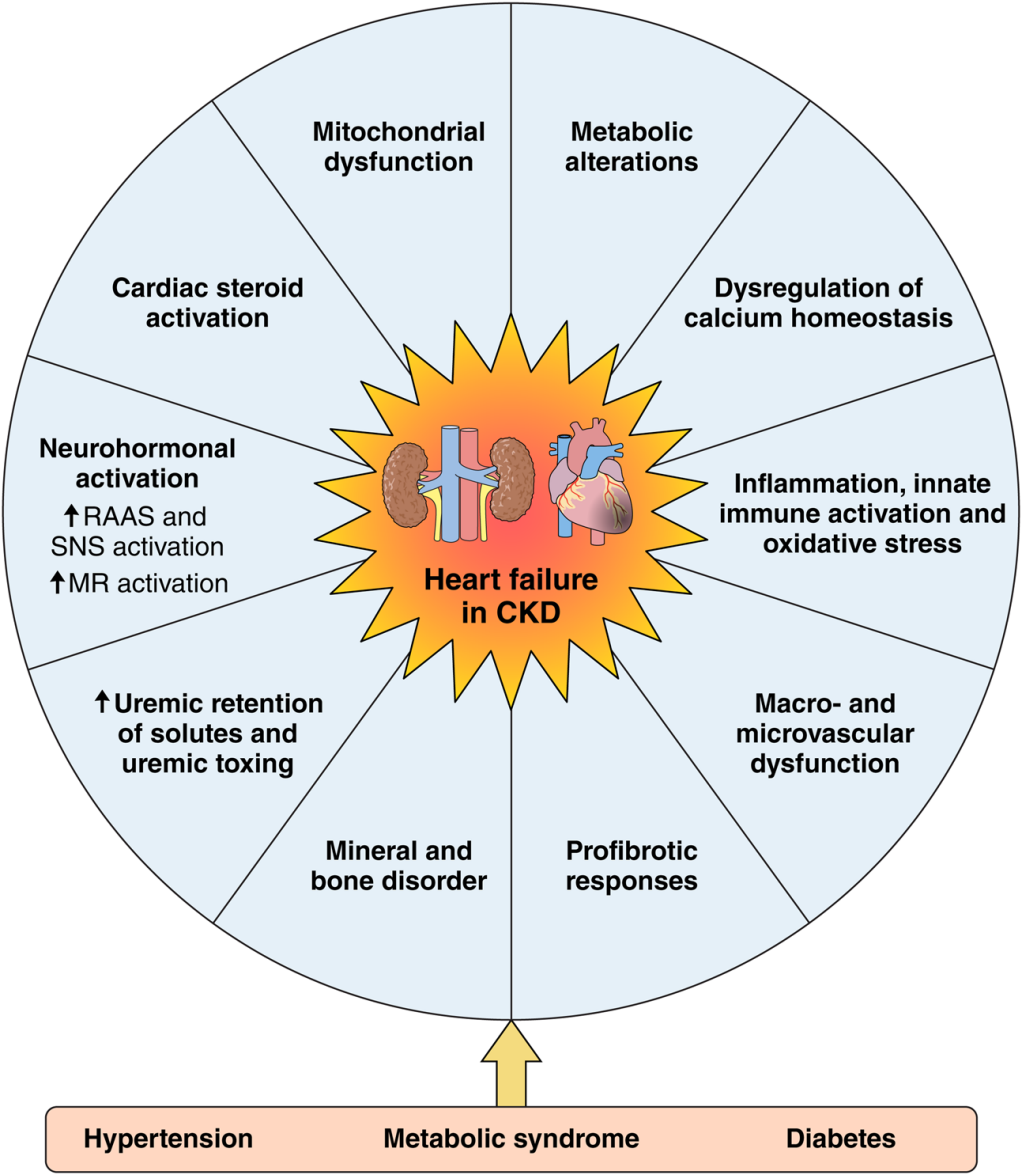
**Pathophysiological mechanisms underlying cardiac remodeling and heart failure in chronic kidney disease (CKD).** The pathophysiological mechanisms underlying cardiac remodeling in CKD are multifactorial. Furthermore, patients with CKD present with comorbidities such as hypertension, metabolic syndrome, and diabetes, which further contribute to cardiovascular pathophysiology and risk. For more information, see text. Illustration credit: Sceyence Studios. MR indicates mineralocorticoid receptor; RAAS, renin-angiotensin-aldosterone system; and SNS, sympathetic nervous system.

Overall, animal models have enabled the identification of pathophysiological mechanisms contributing to cardiac remodeling and HF in CKD.^31,108^ This has highlighted multifactorial contributions, including neurohormonal activation (including the RAAS, the SNS, and MR signaling), cardiac steroid activation, mitochondrial dysfunction, metabolic alterations, and dysregulation of calcium homeostasis in cardiomyocytes. In addition, inflammation, immune activation, oxidative stress, and cellular profibrotic responses in the heart, often in crosstalk with inflammation, contribute to cardiac remodeling in CKD. In addition, uremic retention solutes that accumulate with decreasing kidney filtration function and mineral bone disorders, both presenting also in patients with CKD, further exert detrimental effects on the cardiovascular system by mediating inflammation, fibrosis, and vascular calcification, among others. Together, this triggers cellular and molecular alterations in the vasculature, heart, and blood (including the immune system), contributing to the pathophysiology underlying cardiovascular remodeling and HF in CKD^110^ (Figure 5).

In the following, we review findings of the pathophysiological mechanisms underlying the impact of CKD on the heart as derived from CKD models with uremic cardiomyopathy or cardiac ischemia. We focus on the overall mechanisms and mediators that were identified to contribute to CKD-induced CVD in either mouse or rat models, without a detailed discussion of the method to induce CKD in the applied mouse or rat model, the duration of CKD, the animal strain, and sex, nor the applied diet. For a detailed discussion of how these aspects may impact the presentation of cardiac alterations in CKD mouse models, we refer to our previous reviews.^31,108^ Furthermore, mechanisms of vascular calcification^36^ and cardiac fibrosis^111^ have recently been discussed elsewhere and are, therefore, not addressed separately.

### Cardiac Metabolism and Mitochondrial Dysfunction

#### Alterations in HF

The heart requires a lot of energy in the form of ATP to fuel cardiomyocyte contraction and ion pump activity. In a healthy heart, the oxidation of fatty acids accounts for 70% to 90% of energy production in cardiomyocytes.^112^ Fatty acid transporters mediate the cellular uptake of fatty acids. In the cytoplasm, fatty acids are conjugated to CoA (coenzyme-A) to form fatty acyl-CoA molecules, which are subsequently transported into the mitochondria. Here, fatty acyl-CoA is gradually shortened through fatty acid β-oxidation with simultaneous production of acetyl-CoA, which feeds the tricarboxylic acid cycle (also called the citric acid cycle or the Krebb cycle) for ATP production. Furthermore, nicotinamide adenine dinucleotide and flavin adenine dinucleotide generated during fatty acid β-oxidation and the tricarboxylic acid cycle function as electron donors in the electron transport chain (also referred to as oxidative phosphorylation cycle or respiratory chain) in the mitochondria, which enables a further generation of ATP. Beyond fatty acids, glucose can also be used as a substrate for energy production in the healthy heart (10%–40%), with additional minor contributions to ATP production by oxidizing ketone bodies and amino acids.^112^ Following its cellular uptake by glucose transporters (GLUT1 [glucose LoTransporter protein 1] and GLUT4), glucose is metabolized in the cytoplasm to pyruvate in a process called glycolysis. Pyruvate can then either be converted to acetyl-CoA to fuel the tricarboxylic acid cycle and subsequently oxidative phosphorylation in the mitochondria, or it can be converted to lactate in the cytoplasm. Although the latter pathway is less efficient in energy production, it requires less oxygen.^113^ Overall, the mitochondria contribute to >95% of ATP production in the normoxic heart.

Depending on the required cardiac output, the available substrates, and the (patho)physiological state, the heart can switch its substrate preference.^114^ In stress conditions, the heart initially switches from fatty acid oxidation to glucose utilization.^112,114^ However, the failing heart is characterized by mitochondrial dysfunction, which reduces its capacity for glucose and fatty acid oxidation.^112,114^ As a result, while cardiomyocytes still take up fatty acids, their reduced oxidation in the mitochondria triggers fatty acid accumulation in the cytoplasm, increasing intracellular diacylglycerol and ceramides. Furthermore, the increased uptake of glucose without subsequently increased glucose oxidation in the mitochondria (referred to as the uncoupling of glycolysis and glucose oxidation) may trigger glucose metabolism over the pentose phosphate pathway instead, the hexosamine biosynthetic pathway, or the glycerolipid synthesis pathway.^112^ While the pentose phosphate pathway has been linked to increased superoxide production in HF via NADPH oxidase generation,^115^ the hexosamine biosynthetic pathway synthesizes a substrate for protein glycosylation (O-linked β-N-acetylglucosamine modification), which has been linked to impaired calcium (Ca2^+^)-flux and contractile dysfunctions in cardiomyocytes.^116,117^

Impaired mitochondrial oxidation of glucose and fatty acids triggers a substrate change for energy production, with increased oxidation of ketone bodies, which are increasingly abundant in the plasma of patients with HF.^118^ Supplementation of the ketone body 3-hydroxybutyrate could reduce cardiac remodeling and dysfunction in a mouse model of HF.^119^ However, this most likely cannot compensate sufficiently for reduced myocardial ATP generation in advanced HF, which displays impaired cardiac energetics.^114,120^

Whether adapting to increased glucose uptake and glycolysis in the stressed heart is maladaptive or beneficial has been a matter of debate.^114^ On the one hand, the accumulation of glucose metabolic intermediates or increased glucose metabolism through other pathways could have a negative impact on the cell. On the other hand, cardioprotective effects of increased GLUT1 expression have been reported in cardiac pressure overload.^121^ Others observed GLUT1 overexpression to reduce mitochondrial dysfunction, cardiac fibrosis, apoptosis, and capillary rarefaction, however, without improvement of cardiac dysfunction and with increased cardiac hypertrophy.^122^ This was observed along with increased O-linked β-N-acetylglucosamine modification of calcium cycling proteins, potentially negatively impacting calcium homeostasis.^122^ Similarly, whether restoring fatty acid metabolism could provide cardioprotection has been questioned. In this context, stimulating cardiac fatty acid metabolism and mitochondrial function by activating the estrogen-related receptor as an essential regulator of cardiac metabolism was able to provide cardioprotection in a pressure overload–induced HF mouse model.^123^

In conclusion, the failing heart switches from primarily fatty acid oxidation to glucose utilization for energy supply. However, mitochondrial dysfunction in the stressed heart impairs the mitochondrial oxidation capacity for both fatty acids and glucose intermediates, leading to downstream pathophysiological consequences.

#### Alterations in CKD

Although altered cardiac metabolism in HF has been studied in detail, the impact of CKD on mitochondrial function and especially cardiac metabolism has been less studied. Cardiomyocytes of CKD mice revealed increased oxidative stress, loss of mitochondrial integrity, reduced levels of the mitochondrial electron transport chain complexes I, II, and IV, and impaired mitochondrial respiration capacity.^124^ This could be restored by in vivo overexpression of 2-OGDHL (oxoglutarate dehydrogenase-like), a protein that decreased in CKD mice’s hearts and was downregulated by osteopontin.^124^ Osteopontin is upregulated in CKD and associated with a higher risk of adverse outcomes.^125^ Others showed a chronic uncoupling of mitochondrial respiration of cardiomyocytes of CKD rats, an increased mitochondrial sensitivity to stress-induced permeability pore formation, and increased depolarization rates.^126^

Furthermore, cardiac mitochondrial dysfunction in uremic cardiomyopathy has been observed along with reduced fatty acid oxidation^127,128^ and increased glucose uptake^127^ although this has not been studied yet in much detail. Notably, patients with CKD on hemodialysis display a significant reduction in plasma and muscle L-carnitine levels versus a significant increase in plasma acylcarnitines.^129^ With carnitines being essential in fatty acid metabolism by mediating the transport of long-chain fatty acyl molecules across the inner mitochondrial membrane for β-oxidation, a dysregulation of carnitine levels could potentially contribute to uremic cardiomyopathy via reduced fatty acid metabolism. A rat CKD model with moderate cardiac hypertrophy revealed reduced free carnitine versus an increased ratio of acyl-carnitine/free carnitine in serum; however, it did not display altered carnitine levels in the heart.^130^ Nonetheless, the same group revealed increased glucose versus reduced fatty acid (palmitate) utilization and reduced cardiac expression of the fatty acid transporter CD36 in the hypertrophic heart, though without any impact on cardiac function.^127^ Chronic L-carnitine supplementation abolished CKD-induced hypertrophy in this CKD model, along with a reduction in glucose consumption.^131^ A causative role for decreased myocardial fatty acid metabolism in cardiac damage in CKD was also suggested based on an IRF1 (interferon regulatory factor 1)-mediated decrease in PGC1α expression, a central regulator of mitochondrial biogenesis and oxidative phosphorylation, in hearts of CKD mice, with genetic deficiency of IRF1 protective against high phosphate-induced cardiac alterations in vivo.^128^ In vitro, high phosphate levels, as present in patients with advanced CKD,^132^ downregulated PGC1α expression in cardiomyocytes and in parallel induced mitochondrial dysfunction and increased ROS production.^128^

By facilitating glucose uptake, insulin directly impacts cardiomyocyte metabolism. In addition, insulin triggers cell signaling. Insulin resistance (ie, the inability to increase cellular glucose uptake upon insulin stimulation) and altered insulin signaling in cardiomyocytes have been linked to cardiac hypertrophy, fibrosis, and dysfunction in metabolic diseases.^133^ This can be triggered both through the metabolic and the pleiotropic effects of insulin, the latter of which may be further upregulated upon insulin resistance by reactive hyperinsulinemia as an attempt to maintain serum glucose levels.^134^ The protein kinase AKT plays a crucial role in the signaling pathways downstream of insulin and its detrimental action in cardiomyopathy.^134^ Insulin resistance has been observed in CKD animal models.^135,136^ These revealed a loss of the cardioprotective effects of insulin, which the insulin sensitizer rosiglitazone could improve.^136^ Insulin resistance is also observed in patients with CKD^137,138^ although data on the relation with CKD progression and cardiovascular outcome in CKD are inconsistent.^138,139^

In conclusion, in conditions accompanied by uremic cardiomyopathy, cardiac mitochondrial dysfunction has also been observed along with reduced fatty acid oxidation and increased glucose uptake. However, the underlying mechanisms and their causal contribution to CVD risk in CKD require further clarification.

#### Central Regulators of Cellular Metabolism and Cardioprotective Drugs Impacting Metabolism in HF and CKD

Important regulatory pathways of cardiomyocyte metabolism include mTOR (mammalian target of rapamycin) and AMPK (AMP-activated protein kinase), which have a detrimental or cardioprotective effect on the heart, respectively.^140,141^ Furthermore, novel cardioprotective drugs targeting cell metabolism have been developed with strong cardioprotective effects in HF, including SGLT2is (sodium-glucose co-transporter-2 inhibitors) and GLP-1R (glucagon-like peptide-1 receptor) agonists. Whereas the underlying mechanisms associated with these regulators and drugs have been intensively studied in relation to HF, few studies have been performed on HF in CKD.

The serine/threonine kinase mTOR is a member of the PIKK (phosphoinositide 3-kinase-related kinase) superfamily and part of the mTORC1 (mTOR complex 1), a nutrient-sensing protein complex that has been linked to oxidative stress. mTORC1 also downregulates autophagy and controls cholesterol and fatty acid metabolism, glycolysis, and mitochondrial biogenesis.^140^ More specifically, mTORC1 promotes glycolysis over fatty acid oxidation via induction of HIF-1α.^140^ Although the complete absence of mTORC1 has detrimental effects on the heart,^142^ a partial inhibition of mTORC1 has revealed cardioprotective effects in the presence of cardiac overload, ischemia, and metabolic stress.^140^ In the same line, mTOR signaling was shown to contribute to CKD-induced cardiac hypertrophy and fibrosis, as revealed upon blocking mTORC1 with rapamycin.^143,144^

In contrast to mTOR, AMPK is cardioprotective, also in HF.^141^ AMPK is a nutrient sensor that is activated in response to stress to mitigate imbalances between energy supply and demand. AMPK activation supports mitochondrial function and biogenesis and can, thereby, increase the oxidation of both glucose and fatty acids.^141^ AMPK also has anti-inflammatory and antioxidant effects; it can reduce endoplasmic reticulum stress, in part mediated by the inhibition of mTORC1. Furthermore, AMPK activates autophagy and counteracts cardiac fibrosis.^141^ In a CKD mouse model, increased MI-/resistive index (RI)-induced cardiac damage was observed along with reduced AMPK signaling.^145^

SGLT2 (sodium-glucose co-transporter-2) regulates glucose reabsorption in the proximal tubule, with SGLT2is (gliflozins) increasing glucose excretion and, thereby, reducing blood glucose levels. Overall, SGLT2i provides protection for both the kidney, vasculature, and heart and reduces HF hospitalizations and kidney function decline, both in patients with type 2 diabetes and without.^146^ Mechanistically, the cellular and molecular mechanisms underlying the protective effects of SGLT2i are multifactorial, go beyond increased glucose excretion and natriuresis at the level of the tubules, and are even partly independent of SGLT2 effects as proven in mice lacking SGLT2^147^ (see the study by Preda et al^146^ for a recent detailed discussion). In short, SGLT2i has beneficial effects on the macrovasculature including the endothelium and also counteracts microvascular dysfunction. Furthermore, they are anti-inflammatory, reduce oxidative stress by increasing the bioavailability of nitric oxide, maintain autophagic flux, and protect from mitochondrial stress and dysfunction. Also, SGLT2i has beneficial metabolic effects including reduced insulin resistance and enhanced oxidation of both glucose and long fatty acids independently of insulin levels. In addition, enhanced ketone body production by SGLT2i may contribute to protective effects in relation to HF.^148^ SGLT2i also mediates improved Ca^2^^+^ homeostasis and sodium currents in cardiomyocytes and, thereby, counteracts contractile dysfunctions. They also supported cardioprotective signaling by AMPK^146^ and could reduce the infarct size upon MI by increasing parasympathetic activity.^149^ Specifically, in CKD conditions, the SGLT2i empagliflozin was shown to counteract a CKD-induced reduction in protective endothelial-cardiomyocyte crosstalk.^150^ Studies on the mechanistic impact of SGLT2i in uremic cardiomyopathy and associated HF are awaited.

Furthermore, GLP-1R agonists have cardioprotective effects, including reducing hospitalization rates for HF, as recently discussed in detail elsewhere.^151^ GLP-1R agonists increase insulin secretion, reduce hunger, and delay gastric emptying. As a result, they reduce body weight, glucose levels, and obesity. They also reduce blood pressure and inflammation.^151^ With GLP-1R only expressed to a low level in the heart, cardioprotective effects are most likely a combination of direct and indirect effects. Concerning HF, this includes reduced cardiac inflammation, increased cardioprotective AMPK signaling, and increased myocardial glucose metabolism, the latter probably an indirect effect due to increased insulin secretion.^151^ To our knowledge, no studies specifically studied the impact of GLP-1R agonists on uremic cardiomyopathy and associated HF.

In summary, the nutrient sensors mTOR and AMPK have been linked with cardiodetrimental and cardioprotective functions, respectively, in the context of CKD. Furthermore, SGLT2i and GLP-1R agonists are cardioprotective but have been largely understudied in uremic cardiomyopathy up until now. Therefore, additional studies are required to explore their cardioprotective potential, specifically in CKD, and to reveal the underlying mechanisms, including a potential impact on cell metabolism and inflammation.

### Calcium Mishandling

Uremic hearts show signatures of a disturbed myocardial Ca^2^^+^-flux.^152–155^ This has been observed along with a higher sensitivity of ryanodine receptor channels to Ca2^+152,154^ (with these channels mediating intracellular Ca^2^^+^ entry that triggers cell contraction) versus reduced levels and activity of the SERCA (sarcoplasmic reticulum calcium ATPase)^152^ (which facilitates muscular relaxation through its role in cellular Ca^2^^+^ homeostasis). Disturbed Ca^2^^+^-fluxes could be reduced by enhancing levels of the cardioprotective Klotho^153,156^ or by inhibiting the multifunctional Ca^2^^+^ and CamKII (calmodulin-dependent protein kinase II),^155^ a kinase known to regulate intracellular Ca^2^^+^ levels and excitation-contraction coupling in cardiomyocytes. A disturbed Ca^2^^+^ homeostasis in cardiomyocytes of CKD mice could also be improved by blocking the proinflammatory innate immune receptor NOD1 (nucleotide-binding oligomerization domain-containing protein 1) or its downstream kinase RIP2 (receptor-interacting serine/threonine protein kinase 2).^154^

### Neurohormonal Signaling: Activation of the RAAS, the SNS, and Mineralocorticoid Receptor (MR) Signaling

Patients with CKD show an increased activation of the RAAS, the SNS, and the MR.^85,99,157–159^ RAAS and MR activations are crucial in regulating blood pressure, electrolyte and fluid balance, and vascular resistance.^158,160^ Along with RAAS activation, also activation of the SNS, with the release of neurotransmitters such as norepinephrine, contributes to hypertension, vasoconstriction, vascular stiffness, and endothelial dysfunction.^157^ The activation of these pathways contributes to uremic cardiomyopathy and cardiovascular risk in CKD, at least partially also independent of blood pressure, as discussed in more detail in the following.

#### Renin-Angiotensin-Aldosterone System

Concerning the RAAS, administration of ARBs or ACE inhibitors in CKD animal models could reduce the extent of cardiac remodeling, including cardiac hypertrophy, fibrosis, and inflammation,^161–165^ at least partly independent of blood pressure reductions.^161,163,165^ Also, a role for excessive renin production was revealed in impaired postinfarction cardiac remodeling in CKD, independent of blood pressure or kidney function.^166^ More recently, blockade of the (pro)renin receptor, which can activate both AngII-dependent and independent pathways upon binding (pro)renin,^167^ improved CKD-induced cardiac hypertrophy and fibrosis.^168^

#### Sympathetic Nervous System

Activation of the SNS is a natural stress response, but overactivation can contribute to cellular and organ damage. The cardiac SNS depends on neurotransmitters such as norepinephrine, dopamine, and epinephrine to regulate heart rate, conduction velocity, and myocardial contraction–relaxation responses, and cardiac SNS hyperactivation contributes to detrimental cardiac remodeling and HF.^169,170^ The role of the SNS in CKD-heart crosstalk was revealed by blocking β-adrenergic receptor signaling in CKD rats, which reduced cardiac fibrosis and hypertrophy, either with^171^ or without^172^ simultaneous blood pressure effects. The beta blockade also reduced the CKD-induced activation of CaMKII as a crucial regulator of Ca^2^^+^ signaling in the heart.^172^

Furthermore, it could be shown that both CKD and HF trigger kidney afferent (sensory) nerves, which then induce a neuronal response in the brain that subsequently drives SNS activation in the kidney (for both the CKD and HF model) and the heart (for the HF model), associated with organ fibrosis and dysfunction.^173^ A role for RAAS was identified in mediating the communication of initial kidney or cardiac damage and associated kidney afferent nerve activation with the brain neural circuit.^173^ Despite focusing on SNS activation as a mediator of HF crosstalk with secondary kidney damage, the study did not address the reciprocal impact of kidney damage on the heart.^173^ However, another study did show a beneficial reduction of sympathetic nerve activity by renal denervation on atrial fibrillation vulnerability in CKD.^174^ In a rat model, CKD was shown to induce left atrial structural changes with increased cardiac interstitial fibrosis and sympathetic innervation along with detrimental electrophysiological responses. These features of adverse cardiac remodeling and function could be counteracted by renal denervation, independent of blood pressure and kidney function.^174^ This aligns with findings in clinical studies in which renal denervation in patients with persistent hypertension positively influenced left atrial remodeling independently of blood pressure or heart rate effects.^175^ Complementary studies in hypertensive rats with metabolic syndrome demonstrated a similar protective effect of renal denervation on atrial remodeling with reduced levels of soluble RAGE (receptor for advanced glycation endproducts) and cardiac inflammation, reduced cardiac fibrosis, and reduced left atrial hypertrophy.^176^

#### Mineralocorticoid Receptor (MR)

Overactivation of MR signaling increases sodium retention and hypertension, as well as systemic inflammation, LV hypertrophy, myocardial fibrosis, and cardiac dysfunction,^102,103,177^ as shown by the beneficial effects upon application of the MR antagonist finerenone in CKD.^177^ Also here, cardioprotective effects were at least in part blood pressure–independent, as demonstrated for the steroidal MR antagonist spironolactone in relation to CKD-induced systemic and vascular inflammation^178^ and for the nonsteroidal MR antagonist finerenone in relation to CKD-induced cardiac dysfunction and fibrosis.^177,179^

In summary, RAAS, SNS, and MR signaling are increased in CKD and contribute to increased cardiovascular remodeling through blood pressure–dependent and blood pressure–independent effects.

### Cardiac Steroids

The accumulation of cardiac steroids in CKD has been causally linked with pathological cardiac remodeling, increasing cardiac hypertrophy and fibrosis, as well as cardiac dysfunction.^180^ Cardiac steroids can be categorized into cardenolides (eg, digoxin) and bufadienolides (eg, marinobufagenin) and are strong inhibitors of the transmembrane NKA (Na^+^/K^+^-ATPase) pump. NKA pumps regulate Na^+^ and K^+^ gradients by exchanging intracellular Na^+^ for extracellular K^+^, thereby impacting basic cellular homeostasis within the cardiovascular system, affecting heart rate, blood pressure, and cardiac contraction, among others.^180^ Accordingly, dysregulation of NKA can trigger cardiomyocyte dysfunction.^180^ Mechanistically, the cardiac steroid marinobufagenin, which is increased in patients with CKD, was shown to bind Na^+^/K^+^-ATPase and trigger detrimental cell signaling in cardiomyocytes associated with ROS production.^181^ It also triggered ECM (extracellular matrix) production in fibroblasts in vitro.^182^ In vivo, marinobufagenin infusion was associated with reduced SERCA expression, disturbed Ca^2^^+^-fluxes, and diastolic dysfunction.^181^ In contrast, immunization against marinobufagenin reduced cardiac fibrosis, hypertrophy, and dysfunction in a CKD animal model, independently of blood pressure.^181,182^ Marinobufagenin blockade could also restore CKD-induced defects in vascular relaxation and reduce aortic fibrosis.^183^

In summary, increased levels of cardiac steroids contribute to pathological remodeling of the heart in CKD, as revealed in animal models.

### Inflammation, Oxidative Stress, and Immune Activation

CKD induces low-grade inflammation at a systemic level and in the vasculature and heart (as discussed in more detail in the studies by Junho et al^31^ and Baaten et al^35^). This includes the upregulation of proinflammatory molecules such as cytokines, chemokines, and hs-CRP, as well as activating proinflammatory pathways (eg, NF-κB signaling and the NLRP3 inflammasome). This could also be observed in animal models of CKD, with altered oxidative stress responses detected in the heart^108,184^ and increased inflammation observed at the systemic and organ levels.^31,108,185^

The proinflammatory cytokine TNFα was shown to drive cardiomyocyte hypertrophy in vitro, and TNFα inhibition in vivo could, in part, restore the reduced ejection fraction in mice with CKD induced by either subtotal nephrectomy or ischemia/reperfusion injury with contralateral nephrectomy.^186^ LV single-nucleus RNA sequencing also revealed signatures of cardiac vascular inflammation in these CKD mice.^186^ Others could similarly reveal a causal role for inflammation in uremic cardiomyopathy by directly blocking key inflammatory pathways. For example, blocking the IL-6 receptor,^187^ TLR 4,^188^ or HDAC6 (histone deacetylase 6)-mediated inflammation^189^ reduced cardiac inflammation and fibrosis in CKD rodent models. Conversely, enforcing inflammation in CKD mice by overexpression of the acute phase calgranulin proteins S100A8/A9/A12 (S100 family of calcium-binding “alarmin” proteins: calgranulin A/ calgranulin B/ calgranulin C) induced cardiac hypertrophy, cardiac FGF23 (fibroblast growth factor 23) expression, and diastolic dysfunction via the receptor for advanced glycation end products (RAGE), with proinflammatory stimuli triggering FGF23 expression in cardiac fibroblasts.^190^ Furthermore, a role was revealed for mitochondrial stress-induced activation of the STING (stimulator of interferon genes) pathway in CKD-induced hypertrophy through metabolic alterations: activation of the STING pathway increased proinflammatory NF-κB signaling in myocardial tissue of CKD mice. It, thereby, enhanced the biosynthesis of the polyamine putrescine, which could be shown to contribute to CKD-induced hypertrophy in vivo.^191^

Reduction of oxidative stress had cardioprotective effects in uremic cardiomyopathy.^192–194^ For example, blocking the oxidant amplification function of the Na/K-ATPase or inducing HO-1 (heme oxygenase-1) in CKD mice improved cardiac remodeling and dysfunction.^193^ Also, blocking endoplasmic reticulum stress reduced kidney and cardiac dysfunction in CKD mice,^194^ and inhibiting the NADPH oxidase reduced cardiac oxidative stress, remodeling, and dysfunction in CKD rats.^195^ Furthermore, a role for NOS (nitric oxide synthase)–mediated superoxide production could be shown in relation to diastolic dysfunction in a hypertensive mouse model with uninephrectomy.^196^ Endothelial mitochondrial stress and ROS production induced by CKD serum also reduced nitric oxide bioavailability to cocultured cardiomyocytes, interfering with endothelial support of cardiomyocyte contraction and relaxation.^150^ The SGLT2i empagliflozin could reduce CKD-induced endothelial ROS production and, thereby, restore a protective endothelial-cardiomyocyte crosstalk.^150^

Deficiency of the antioxidant enzyme glutathione peroxidase-3 induced premature mortality in CKD mice, which was associated with increased platelet activation and the formation of intracardiac platelet microthrombi in CKD (but not non-CKD) mice.^197^

In relation to immune cells, kidney injury triggered chemokine CCL8 expression and inflammatory cell recruitment in the heart, and CCL8 blockade reduced cardiac inflammatory cell infiltration, fibrosis, and dysfunction.^198^ Also, the chemokine CXCL10 and the chemokine receptor CCR2 were shown to contribute to macrophage infiltration in the uremic heart.^199^ Furthermore, T cells infiltrate the heart in CKD and contribute to diastolic dysfunction.^200^ Platelet depletion improved cardiac remodeling in CKD, with a detrimental role revealed for MMP-7 (matrix metalloproteinase-7) in platelet-activated macrophages.^201^

Oxidative stress and proinflammatory responses also contribute to cardiac damage in the ischemic heart in CKD. Endoplasmic reticulum stress-mediated apoptosis contributed to a CKD-associated increase in infarction size and cardiac dysfunction following cardiac ischemia-reperfusion injury (MI/RI).^202^ Enhanced MI-/RI-induced apoptosis, infarction size, and cardiac dysfunction in CKD mice reduced cardioprotective adiponectin/AMPK signaling in CKD.^145^ Inflammation is also involved in increased atrial fibrillation risk in CKD, with a detrimental role shown for the NLRP3 inflammasome.^203,204^

In summary, oxidative stress, inflammation, and immune cell activation contribute to increased cardiovascular risk in CKD, shown in the context of uremic cardiomyopathy, atrial fibrillation, and the ischemic heart.

### Mineral and Bone Disorders

Mineral and bone disorders emerge as severe complications of CKD, and biomarkers of mineral and bone disorders have also been implicated as prognostic and contributing factors in increased cardiovascular risk in CKD.^205,206^ Mineral and bone disorder biomarkers include increased levels of phosphate, PTH (parathyroid hormone), and the phosphaturic hormone FGF23 versus reduced levels of vitamin D and Klotho, among others.^206^ High serum phosphate in CKD drives vascular calcification and, thereby, arterial stiffness and cardiovascular risk.^36^ Furthermore, high phosphate levels contributed to oxidative stress, atrial fibrosis, and atrial fibrillation vulnerability in mice, even without additional CKD induction, with high phosphate triggering profibrotic responses in fibroblasts via inflammation and oxidative stress pathways.^207^ In line, the phosphate binder sevelamer reduced aortic stiffness, diastolic dysfunction, and LV hypertrophy in CKD mice, with early beneficial actions independent of its FGF23-lowering effects.^208^ Also, the iron-based phosphate binder ferric citrate improved CKD-induced cardiac dysfunction independently of its effect on CKD progression, attributed to corrections of FGF23 and phosphate excess and potential restoration of CKD-associated anemia and iron deficiency.^209^

FGF23 was shown to drive cardiomyocyte hypertrophy^210^ through FGFR4 (FGF receptor isoform 4), which, in a CKD mouse model, was further supported by heparin (which is routinely applied to patients with CKD on dialysis) but could be counteracted by Klotho.^211^ FGF23 can also promote cardiac rhythm and contractile alterations^212,213^ and was shown to contribute to microvascular dysfunction with reduced endothelium-dependent vasodilation in a CKD mouse model.^214^ Also, the cardioprotective effects of DMP1 (dentin matrix protein 1) in a CKD mouse model were suggested to be mediated by a lowering of FGF23 despite increased serum phosphate and impaired kidney function.^215^

αKlotho is a transmembrane co-receptor for FGF23 and, thereby, involved in regulating phosphor homeostasis, but it is also present as soluble Klotho through which it can act independently of FGF23. In contrast to FGF23, αKlotho has cardioprotective effects in uremic cardiomyopathy^216–219^ but is reduced in CKD.^31^ αKlotho reduces vascular calcification in CKD^220^ and also counteracts cardiac hypertrophy, cardiac fibrosis,^216,217^ and disturbed myocardial Ca^2^^+^-fluxes in uremic cardiomyopathy.^153^ Furthermore, it has anti-inflammatory effects in CKD on both systemic and local level.^221,222^ αKlotho also provided protection from the detrimental effects of the uremic toxins indoxyl sulfate (IS) and p-Cresyl sulfate (pCS) on the heart.^223–225^

In summary, mineral and bone disorders are an important complication of CKD. Some of its biomarkers contribute to the pathophysiology of CVD in CKD, including vascular calcification and stiffness, uremic cardiomyopathy, and atrial fibrillation vulnerability.

### Uremic Toxins

In the class of protein-bound uremic toxins, IS, pCS, and indole-3-acetic acid all have been associated with cardiovascular risk in patients with CKD.^226–229^ At the mechanistic level, they exert pathophysiological effects on the cardiovascular system, for example, by triggering inflammation and oxidative stress, endothelial dysfunction, and vascular calcification.^230–232^ Also, trimethylamine N‐oxide (TMAO) is classified as a uremic toxin and is associated with increased cardiovascular risk in CKD.^233,234^

IS, pCS, and indole-3-acetic acid were also shown to worsen uremic cardiomyopathy in CKD animal models. IS-induced myocardial inflammation is characterized by NLRP3 inflammasome activation, proinflammatory NF-κB activation, and cytokine expression. This was observed along with cardiac fibrosis, hypertrophy, and dysfunction.^235^ Also, IS induced cardiac hypertrophy in mice by inducing FGF23/FGFR4 signaling.^219^ Furthermore, a role for TSP1 (thrombospondin 1) in IS-induced cardiac hypertrophy and inflammation was revealed, with TSP1 contributing to LV hypertrophy, fibrosis, and dysfunction in CKD mice.^236^ IS also induced cannabinoid receptor type 1 in cardiomyocytes, and inhibiting the latter reduced profibrotic responses in cardiomyocytes in vitro and in the myocardium of CKD mice.^237^ pCS enforced cardiac dysfunction in CKD animals by enhancing cardiomyocyte apoptosis along with oxidative stress, at least partly via NADPH oxidase activity and ROS production.^238^ Indole-3-acetic acid induced oxidative stress, cardiac hypertrophy, and cardiac profibrotic responses in CKD animal models.^239,240^

On the level of the vasculature, IS, pCS, and indole-3-acetic acid, as well as other uremic toxins, have all been linked to endothelial inflammation and oxidative stress.^232^ Furthermore, multiple uremic toxins accumulating in CKD have been linked to reduced vascular relaxation, as shown for IS,^241^ advanced glycation end products, and FGF23.^232^

TMAO is produced mainly in the gut by the metabolism of L-carnitine, choline, and phosphatidylcholine with the help of gut microbiota. High concentrations of TMAO have been linked with HF development through a role in cardiac hypertrophy and fibrosis, mitochondrial dysfunction, and disturbances in Ca^2^^+^-fluxes and cardiomyocyte contractility.^234^ TMAO is stage-dependently increased in CKD, potentially through a contribution of gut dysbiosis in CKD.^242^ Thus, TMAO could form an additional link between CKD and HF.

In addition to direct cellular effects, uremic toxins can induce posttranslational modifications of proteins and peptides, with, for example, carbamylation and guanidinylation highly detected in patients with CKD. By impacting protein structure, localization, or interaction with other proteins, these posttranslational modifications can trigger alterations in protein function and, thereby, induce pathophysiological effects that contribute to CKD-associated CVD, as we discussed in detail elsewhere.^243^ For example, carbamylated low-density lipoprotein, carbamylated sortilin, and guanidinylated fibrinogen are increased in patients with CKD and associated with increased cardiovascular risk in CKD, with a mechanistic involvement identified in increased inflammation and oxidative stress, vascular calcification, and prothrombotic responses, respectively.^243^

In summary, uremic toxins and associated posttranslational modifications are important causal contributors to cardiovascular risk in CKD and, therefore, interesting potential targets of future therapeutic and preventive strategies.

### Gut Dysbiosis

The gut microbiome performs various tasks, such as food digestion, development of host immunity, control of gut endocrine function, and production of various substances. It can, therefore, be considered a metabolically active endogenous organ.^244^ The gut microbiome also contributes to the production of uremic toxins, such as IS and pCS. The bacterial proteolytic fermentation process accounts for most potentially toxic end products.^245^ Thus, most gut-derived toxins are nitrogenous compounds. For example, IS is generated from tryptophan that is converted into indole by gut microbial tryptophanase enzymes and subsequently transferred to the liver where it is hydroxylated and sulfated by cytochrome P450s and sulfotransferase enzymes.^246^ pCS is generated by conversion of tyrosine to p-cresol by microbial enzymes, which is then subsequently transformed into pCS by hepatic sulfotransferases.^247^

CKD results in several structural and functional gastrointestinal changes, such as an impaired intestinal barrier, impaired digestion, and an impaired gut microbiome. Notably, these microbiome changes result in a shift from producing beneficial short-chain fatty acids to an increased production of uremic toxins.^248^ The interaction of the microbiome and CKD has recently nicely been reviewed elsewhere. Initial intervention studies have also shown promising effects of probiotics on CKD progression. For example, the oral administration of Lactobacillus casei Zhang delayed the progression of CKD in mice and slowed the decline in kidney function in patients with CKD stages 3 to 5. However, long-term studies are still required to confirm these beneficial effects.

In summary, CKD development triggers gut dysbiosis, resulting in increased production of uremic toxins, generating a vicious cycle. Therefore, adjusting and improving gut health are valuable therapeutic approaches for CKD.

### Macrovascular and Microvascular Dysfunction in CKD

CKD impairs endothelium-dependent vasodilation of the peripheral microvasculature, as shown in CKD mouse models.^214,249^ This could be restored by treatment with epoxide hydrolase, which metabolizes the vasodilatory epoxyeicosatrienoic acids produced by endothelial cells and which, in parallel, also improved CKD-induced cardiac hypertrophy, fibrosis, and dysfunction independently of kidney protection or blood pressure.^249^ Beyond peripheral microvascular dysfunction, myocardial microvascular dysfunction has been shown in CKD mice, with a potential role for reduced cardiac HIF1α levels and, thereby, reduced hypoxia-driven angiogenesis.^64^ Microvascular dysfunction included capillary rarefaction, as well as reduced blood flow velocity, vascular tone, and oxygen uptake.^64^ CKD rats revealed a capillary rarefaction in the heart along with an increased infarction size following myocardial ischemia-reperfusion injury. Furthermore, myocardial oxygen delivery was impaired in a comorbid swine model with combined hyperglycemia, hypercholesterolemia, diabetes, and CKD.^250^ The hearts revealed an increased oxidative environment in comorbid conditions, reduced nitric oxide bioavailability, and impaired endothelium-dependent vasodilation of the coronary microvasculature in the comorbid swine. This vascular dysfunction could be improved by in vivo ROS scavenging.^250^

In summary, both macrovascular and microvascular dysfunctions are present in CKD. However, our knowledge of the main molecular contributors and regulators is still limited, necessitating additional studies for clarification.

## Hypertension as the Key Comorbidity of CKD and HF: Clinical Characteristics of Hypertensive Heart Disease and Genetic Predisposition

### Hypertension as a Key Cause of CKD

Hypertension is a well-established risk factor for the development and progression of both CKD and HF. An analysis of the Framingham Offspring Study provided an accurate estimation of the lifetime risk of CKD, revealing that individuals with hypertension at baseline have a significantly higher lifetime risk of developing CKD. Specifically, the cumulative lifetime risk of CKD for individuals with hypertension was 50.2% (95% CI, 46.1–54.3) compared with 34.2% (95% CI, 29.4–39.0) for those without any risk factors.^251^ Epidemiological studies have further demonstrated that a strong association between hypertension and kidney disease extends to ESRD. Indeed, the MRFIT (Multiple Risk Factor Intervention) study^252^ showed a progressive increase in the relative risk of ESRD with rising blood pressure. For example, the relative risk of ESRD for men with stage 1 hypertension (systolic blood pressure, 140–159 mm Hg) was 2.8, increasing to 5.0 at stage 2 (160–179 mm Hg), 8.4 at stage 3 (180–209 mm Hg), and 12.4 at stage 4 (≥210 mm Hg) compared with those with optimal systolic blood pressure (120 mm Hg). In the Kaiser Permanente cohort (with 316 675 adults with normal kidney function), there was also a dose-response relationship between increasing blood pressure and ESRD risk, with an adjusted relative risk increasing progressively from 1.62 for blood pressure of 120 to 129/80 to 84 mm Hg to 4.25 for blood pressure ≥210/120 mm Hg.^253^ Trials comparing different blood pressure targets have shown that intensive blood pressure reduction can slow CKD progression, particularly in patients with proteinuria. In the MDRD (Modification of Diet in Renal Disease) study, intensive blood pressure control led to significantly slower CKD progression in patients with proteinuria.^254^ The AASK (African American Study of Kidney Disease and Hypertension) study showed that intensive blood pressure control did not significantly alter the pace of GFR decline compared with the standard group, but benefits were apparent in patients with higher proteinuria levels.^255^ Pooled data from the MDRD and AASK studies demonstrated that intensive blood pressure control was protective against ESRD in specific subgroups, such as those with baseline proteinuria ≥0.44 g/g, low GFR (<30 mL/min per 1.73 m²), older age, and those with obesity (ie, body mass index ≥30 kg/m²).^256^

### Hypertension as a Key Cause of HF

Hypertension is also a major driver of the development and progression of HF, particularly in patients with HFpEF. The prevalence of hypertension among patients with HFpEF ranges from 55% to 90%.^257,258^ In the OPTIMIZE-HF (Organized Program to Initiate Lifesaving Treatment in Hospitalized Patients With Heart Failure) registry, hypertension was identified as the underlying cause of HF in 17% of patients with HFrEF, 22% in those with HF with mildly reduced ejection fraction, and 31% in those with HFpEF.^259^ Similarly, the Swedish HF registry reported that 56% of patients with HFrEF, 64% with HF with mildly reduced ejection fraction, and 72% with HFpEF had a history of hypertension.^260^ The incidence of HFpEF is rising in developed countries, primarily driven by an aging population and increasing rates of uncontrolled hypertension.^261^

While the lifetime risk of developing HFpEF is notably high, with an incidence rate of 1 in 10 adults aged ≥45 years,^262^ this risk is nearly twice as high in individuals with hypertension compared with those with normal blood pressure.^263,264^

### Hypertensive Heart Disease

Hypertensive heart disease represents a spectrum of structural and functional cardiac changes in response to persistently elevated blood pressure.^265^ The prevalence of hypertensive heart disease increased by >50% from 1990 to 2017 in developing countries, representing a growing share of the global CVD burden.^266^ The progression of hypertensive heart disease can be classified into 4 main phases: (1) initial diastolic dysfunction, (2) development of LV hypertrophy, (3) advanced diastolic dysfunction with clinical symptoms, and (4) eventual systolic dysfunction.^267^

#### Phase 1: Initial Diastolic Dysfunction (Stage A)

In the early stages of hypertensive heart disease, also known as stage A, concentric remodeling occurs. This includes the development of septal bulge and delayed myocardial relaxation. LV compliance is relatively preserved during this phase, and functional changes predominate without significant structural alterations.

#### Phase 2: Development of LV Hypertrophy (Stage B)

As chronic hypertension progresses, the heart develops concentric hypertrophy of the LV. The prevalence of hypertension-driven LV hypertrophy reaches ≈30% in hypertensive individuals. LV hypertrophy is a structural adaptation to sustained pressure overload, resulting in increased myocardial wall thickness and decreased compliance, exacerbating diastolic dysfunction. Beyond the increase in LV mass, echocardiography often reports an early onset of left atrial enlargement (which signifies rising filling pressures) at this stage. In further evolution, concentric hypertrophy (from pressure overload) is followed by eccentric hypertrophy (with volume overload) and eventually myocardial fibrosis, as documented by several studies.^265^

#### Phase 3: Advanced Diastolic Dysfunction and Clinical Symptoms (Stage C)

LV concentric hypertrophy precedes HFpEF in nearly 75% of hypertensive patients with early signs of HF.^268^ At this stage, patients begin reporting clinical symptoms due to elevated LV filling pressures and marked stiffness of the myocardium. The progression from concentric hypertrophy due to pressure overload to diastolic dysfunction, then to HFpEF, and ultimately to systolic dysfunction and remodeling, is well documented.^269^

#### Phase 4: Systolic Dysfunction and HFrEF (Stage D)

At this stage, the LV transitions from hypertrophic remodeling to systolic dysfunction. This is characterized by a dilated cardiomyopathy phenotype with a reduced LV ejection fraction, marking the onset of HFrEF. Advanced imaging techniques reveal reductions in longitudinal and radial systolic function, indicating a significant decline in overall LV performance.

Adequate blood pressure control significantly reduces the future risk of HF, that is, optimal blood pressure is associated with a reduction of HF risk by ≈50% in patients with hypertension.^267^ Blood pressure–lowering medications, including those operating through the kidney (ie, diuretics), are the mainstay of treatment of hypertensive heart disease.^270,271^ Indeed, sodium retention is apparent even in asymptomatic LV hypertrophy and contributes to increased preload.^272^

Collectively, these data illustrate the key role of elevated blood pressure in the development and progression of both CKD and HF across the entire clinical spectra of both diseases and further emphasize the central role of blood pressure control in the prevention and treatment of both CKD and HF.

### Genetic Predisposition to CKD, Hypertension, and HF Converges at the Kidney

#### CKD, Hypertension, and HF as Heritable Traits

CKD, HF, and hypertension have all been observed to aggregate in families. For CKD, the heritability estimates range from 25% to 44%, and the heritability of its defining traits, that is, eGFR and urinary ACR, is estimated at 44% and 18%, respectively.^273,274^ Blood pressure is similarly heritable, with estimates of 17% to 52% for systolic blood pressure and 19% to 41% for diastolic blood pressure, varying by population and clinic/ambulatory measurement.^275^ HF heritability is estimated at 26%, with HFrEF being more heritable than HFpEF.^276^ This genetic heritability manifests as the increased risk of these diseases in families. The Lifelines study reported a 3-fold increase in the relative risk of CKD in the first-degree relatives of patients with CKD.^274^ Siblings of hypertensive patients had a recurrent risk of hypertension estimated at ≈1.2 to 1.4 in the BRIGHT (Randomized Trial of Bendamustine-Rituximab or R--CHOP/R-CVP in First-Line Treatment of Indolent NHL or MCL) study,^277^ and the Framingham Offspring Study found that parental history of HF is associated with a 70% increase in HF risk when adjusting for known risk factors.^278^

#### Monogenic Forms of CKD, Hypertension, and HF Exhibit Cardiorenal Phenotypes

Familial aggregation of CKD, HF, and hypertension infrequently has a monogenic cause due to Mendelian inheritance of a highly penetrant rare variant. For CKD stages G3b to G5, up to 30% of adult cases are attributable to Mendelian disorders.^279^ Autosomal dominant polycystic kidney disease is caused by loss-of-function variants in *PKD1* or *PKD2* and accounts for up to 10% of ESRD cases.^280^ Typical of monogenic kidney diseases, mortality in autosomal dominant polycystic kidney disease stems from the high prevalence of cardiovascular complications, with 60% of patients experiencing hypertension before GFR decline and 48% of hypertensive patients exhibiting LV hypertrophy.^281^

Monogenic forms of hypertension are almost exclusively caused by defects in sodium handling in the distal nephron and present as early onset, severe, and resistant hypertensions. For example, gain-of-function variants in genes encoding subunits of the amiloride-sensitive epithelial sodium channel ENaC (epithelial Na+ channel) cause Liddle syndrome through upregulation of ENaC on the surface of tubular epithelium.^282^ Hypertension in Liddle syndrome can lead to both CKD and CVD, including HF.^283^

Monogenic causes of HF include cardiomyopathies driven by pathogenic variants in several cardiac genes, such as *TTN*, causing familial dilated cardiomyopathy.^283^ Infrequently, Mendelian variants in renal genes are implicated in dilated cardiomyopathy and HF. Notably, gain-of-function variants in *RRAGD* (expressed in the thick ascending limb of loop of Henle and distal convoluted tubule) cause salt-wasting tubulopathy and dilated cardiomyopathy, resulting in HF.^284^

#### Overlapping Genetic Architecture and Relationships Between CKD, Hypertension, and HF

In most cases, familial aggregation of CKD, hypertension, and HF reflects polygenic inheritance, implicating many common variants, each with small effects. Large genome-wide association studies (GWAS) have uncovered the identity of genetic variants associated with the risk of CKD, hypertension, and HF and their defining traits. The largest GWAS of eGFR in over 1.2 million participants has identified 424 loci for eGFR (estimated from creatinine), including 348 loci with support from eGFR estimated from cystatin C or blood urea nitrogen.^285^ GWAS of urinary ACR in over 500 000 participants identified 68 genomic loci.^286^ For blood pressure, 2103 loci have now been associated with diastolic blood pressure, systolic blood pressure, and pulse pressure in over 1 million individuals.^287^ The largest GWAS of HF, with 90 000 cases and over 1 million controls, identified 38 loci for HF.^288^ When examined separately, 13 loci were associated with HFrEF and 1 with HFpEF.^276^

GWAS findings have paved the way for the estimation of genetic correlations and facilitated causal inference analyses between CKD, hypertension, and HF. The calculation of genetic correlation leverages GWAS results to quantify the shared genetic background between different phenotypes. Indeed, significant genetic correlations between systolic/diastolic blood pressure and HF (r=0.29/0.21),^289^ alongside systolic/diastolic blood pressure and urinary ACR (r=0.19/0.11), were identified.^286^ In an analysis of 47 309 HF cases and 930 014 controls of European ancestry, Shah et al^289^ found no evidence of a significant genetic correlation between eGFR and HF.

Using genetic variants from GWAS as instruments, Mendelian randomization (MR) assessed causal relationships between these phenotypes. A bidirectional Mendelian randomization (MR) study by Yu et al^290^ analyzed data from 567 460 participants from the CKDGen (Chronic Kidney Disease Genetics) consortium and the UK Biobank and found that better kidney function (measured as an increase in eGFR) significantly reduced blood pressure. The study found no significant causal effect of increased blood pressure on kidney function.^290^ However, using MR with kidney-specific genetic variants as instruments, Eales et al^291^ found a significant causal association between blood pressure and CKD, and blood pressure and urinary ACR. MR analysis by Shah et al^289^ demonstrated a causal relationship between blood pressure and HF risk. Specifically, a 10-mm Hg increase in diastolic blood pressure raised the odds of developing HF by 30% (odds ratio, 1.30; *P*=9.13×10^^⁻^²¹^), while a similar increase in systolic blood pressure increased HF risk by 18% (odds ratio, 1.18; *P*=4.8×10^^⁻^²³^).^289^ Importantly, the effects of hypertension on HF remained significant even after adjusting for coronary artery disease, suggesting that mechanisms beyond coronary artery disease contribute to the increased HF risk associated with elevated blood pressure.

Collectively, these studies demonstrated the existence of a strong genetic component in the predisposition to CKD, HF, and hypertension, defined blood pressure as a causal driver of both CKD and HF, and suggested that both HF and CKD share (at least to some) extent some genetic background.

#### Kidney Gene Expression at the Intersection of CKD, Hypertension, and HF

An accumulating body of evidence indicates that the expression of kidney genes acts as the molecular mediator between DNA variants and predisposition to hypertension and CKD.

Molecular omics studies highlight the kidney’s predominant role as a mediator of genetic susceptibility to hypertension. Eales et al^291^ identified 179 kidney genes with putatively causal effects on blood pressure. The recent analysis of up to 700 kidney transcriptomes increased the catalog of kidney genes with potentially causal effects on blood pressure to 399.^292^ Many multiomics analyses conducted using human kidney tissue uncovered the identity of hundreds of kidney genes whose expressions (or other molecular traits such as DNA methylation and protein abundance) are the likely contributors to genetic regulation of CKD-defining traits.^285,293–301^ There is some overlap (albeit weak) between kidney genes whose expression is associated with both hypertension and CKD-defining traits.^292,299^ Interestingly, integrative omics analyses have also prioritized renal genes at HF risk loci, and some of these genes are well-established contributors to the regulation of blood pressure and human hypertension. For example, *CLCKNA*, a gene encoded by a renal chloride channel was nominated in a transcriptome-wide association study of HF conducted in 8 cardiometabolic tissues.^302^ Rare mutations in *CLCNKA* variants are well-established causes of Bartter syndrome (a monogenic form of low blood pressure),^303^ and a functional *CLCNKA* missense variant has previously been associated with HF, increased blood pressure, and decreased eGFR in several studies.^285,304–307^ *KLHL3*, an upstream regulator of the renal thiazide-sensitive sodium chloride transporter (encoded by *SLC12A3*), was prioritized as an HFrEF locus in the analysis of blood transcriptomes.^289^ *KLHL3* loss-of-function variants cause familial hyperkalemic hypertension (Gordon syndrome, pseudohypoaldosteronism type II), a rare syndrome of Mendelian hypertension.^308^ Through the convergence of plasma proteomics and HF GWAS, decreased circulating levels of aminopeptidase A (encoded by *ENPEP*) were causally associated with increased risk of HF.^288^ Encoded by *ENPEP*, aminopeptidase A shows a high level of expression in the human kidney, whereby it is responsible for conversion of Ang II into Ang III and possibly renal excretion of sodium. Reduced expression of *ENPEP* in the kidney was causally associated with an increase in blood pressure and an increased risk of hypertension and renal sodium excretion.^292^

These studies reveal the causal role of the kidney in the development of CKD and hypertension and highlight its contribution to HF pathogenesis. The common genetic origins of CKD, hypertension, and HF in the form of shared kidney genes operating at the intersection of water-sodium balance and the RAAS further illuminate the importance of these pathways in the development and treatment of these disorders.

## Conclusions

Patients with CKD present an increased risk of HF and over the past decade, multiple studies have contributed to a better understanding of this pathophysiological kidney-heart crosstalk and the interplay with recurrent comorbidities such as hypertension. This revealed that the mechanisms underlying cardiac remodeling in CKD are multifactorial, with a contribution of neurohormonal activation (including the RAAS, the SNS, and MR signaling), cardiac steroid activation, as well as mitochondrial dysfunction, metabolic alterations, and dysregulation of calcium homeostasis. Also, inflammation, immune activation, and oxidative stress, as well as cellular profibrotic responses, contribute to CKD-associated CVD.

Effectively addressing the renal causes of HF requires a comprehensive approach that includes both lifestyle changes and medication, as discussed in more detail recently.^309^ In short, diuretics are essential for controlling fluid overload in patients with CKD,^310^ and medications including ACE inhibitors and ARBs,^311,312^ SGLT2is,^313–315^ and MR antagonists^316^ are essential in managing HF while providing protective effects on the kidneys and lowering blood pressure.^309^ Furthermore, GLP-1R agonists are increasingly gaining clinical importance in the context of HF and kidney damage.^317,318^ As discussed above, both SGLT2is, MR antagonists, and GLP-1R agonists also exert anti-inflammatory effects, further supporting the concept that anti-inflammatory strategies may have a high value in treating CKD and CKD-associated CVD in the future.^70^ This may include the NLRP3/IL-1β/IL-6 pathway, and the ZEUS trial is currently evaluating the impact of IL-6 inhibition by ziltivekimab in patients with CVD, CKD, and inflammation.^81^ Novel strategies may also include targeting innate immune activation although this requires additional mechanistic studies to unravel in more detail the impact of CKD on innate immune cells and subsequent effects on CVD.

The accumulation of uremic toxins in patients with CKD and uremic toxin–induced posttranslational modifications of proteins and peptides in CKD certainly contributes to the induction of these pathophysiological responses. Strategies to adsorb uremic toxins with adsorber particles can reduce uremic toxin concentrations in blood but require further investigation before potential clinical application. Furthermore, strategies to interfere with uremic toxin–induced, pathophysiological posttranslational modifications of proteins and peptides, for example, by scavenging with free amino acids, may provide an interesting therapeutic and preventive approach. Human pilot studies already revealed the potential of amino acid supplementation to reduce the amount of posttranslational modifications in the blood of patients with CKD on dialysis. However, additional studies are required to further optimize these strategies and confirm their safety and efficacy.

Finally, further studies are awaited to reveal additional mechanistic and molecular insights into the impact of CKD on cellular metabolism and energetics, vascular remodeling and endothelial dysfunction, and cardiac remodeling and vulnerability. Here, a combination of multiomics with experimental approaches could further advance insights into pathophysiological crosstalk between the kidney and the heart and could further support the development of additional therapies to reduce HF risk specifically tailored to patients with CKD. By elaborating on the current knowledge of pathophysiological crosstalk of the damaged kidney with the heart, this review aims to support such future endeavors.

## ARTICLE INFORMATION

### Sources of Funding

This work was supported by the German Research Foundation (DFG SFB/TRR219 project ID 322900939 and DFG SFB1382 project ID 403224013), the Interdisciplinary Centre for Clinical Research within the Faculty of Medicine at RWTH Aachen University (to E.P.C. van der Vorst and grant 1-12 to H. Noels), the German Centre for Cardiovascular Research (to E.P.C. van der Vorst and grant DZHK-B23Ex to H. Noels), and by a grant from the European Foundation for the Study of Diabetes-Boehringer Ingelheim (to H. Noels). Additional support was provided by the British Heart Foundation grants (PG/22/10957, PG/19/16/34270, and PG/17/35/33001), Kidney Research UK grants (RP_017_20180302 and RP_013_20190305), British Heart Foundation Centre of Research Excellence (grant RE/24/130017), and National Institute for Health and Care Research (NIHR) Manchester Biomedical Research Centre (grant NIHR203308 to M. Tomaszewski).

### Disclosures

J. Jankowski has given lectures for Bayer and Fresenius Medical Care. In addition, he holds 4 patents on the topic of the article and is the inventor of an additional, already-sold patent to Baxter. N. Marx has given lectures for Bayer, Boehringer Ingelheim, Sanofi-Aventis, MSD (Merck Sharp and Dohme), Bristol Myers Squibb (BMS), AstraZeneca, Lilly, and Novo Nordisk; has received unrestricted research grants from Boehringer Ingelheim; and has served as an advisor for Bayer, Boehringer Ingelheim, Sanofi-Aventis, MSD, BMS, AstraZeneca, and Novo Nordisk. In addition, he served in trial leadership for Boehringer Ingelheim and Novo Nordisk. N. Marx declines all personal compensation from pharma or device companies. The other authors report no conflicts.
